# May the Odds Be Ever in Your Favor: Non-deterministic Mechanisms Diversifying Cell Surface Molecule Expression

**DOI:** 10.3389/fcell.2021.720798

**Published:** 2022-01-11

**Authors:** Donnell L. Williams, Veronica Maria Sikora, Max A. Hammer, Sayali Amin, Taema Brinjikji, Emily K. Brumley, Connor J. Burrows, Paola Michelle Carrillo, Kirin Cromer, Summer J. Edwards, Olivia Emri, Daniel Fergle, M. Jamal Jenkins, Krishangi Kaushik, Daniella D. Maydan, Wrenn Woodard, E. Josephine Clowney

**Affiliations:** ^1^ MCDB 464 – Cellular Diversity in the Immune and Nervous Systems, University of Michigan, Ann Arbor, MI, United States; ^2^ Department of Molecular, Cellular and Developmental Biology, The University of Michigan, Ann Arbor, MI, United States

**Keywords:** monogenic, monoallelic, stochastic gene choice, V(D)J recombination, Dscam, protocadherin, olfactory receptor, antigenic variation

## Abstract

How does the information in the genome program the functions of the wide variety of cells in the body? While the development of biological organisms appears to follow an explicit set of genomic instructions to generate the same outcome each time, many biological mechanisms harness molecular noise to produce variable outcomes. Non-deterministic variation is frequently observed in the diversification of cell surface molecules that give cells their functional properties, and is observed across eukaryotic clades, from single-celled protozoans to mammals. This is particularly evident in immune systems, where random recombination produces millions of antibodies from only a few genes; in nervous systems, where stochastic mechanisms vary the sensory receptors and synaptic matching molecules produced by different neurons; and in microbial antigenic variation. These systems employ overlapping molecular strategies including allelic exclusion, gene silencing by constitutive heterochromatin, targeted double-strand breaks, and competition for limiting enhancers. Here, we describe and compare five stochastic molecular mechanisms that produce variety in pathogen coat proteins and in the cell surface receptors of animal immune and neuronal cells, with an emphasis on the utility of non-deterministic variation.

## Introduction

Despite the shocking complexity of eukaryotic life, eukaryotic genomes often contain less than 20,000 protein-coding genes. While most genes are expressed in a deterministic manner, a variety of molecular mechanisms have been discovered that expand the coding capacity of the genome by expressing cell surface molecules in a quasi-random manner. Expression systems that accomplish cell surface molecule diversification make use of genomic rearrangement, RNA splicing, and epigenetic restriction to create a vast array of molecular variants from a limited amount of DNA. In this way, the static information within genomes can generate a wider diversity of cells throughout the body or across unicellular populations.

This diversity is crucial for the proper functioning of many different biological systems. The immune system, for example, relies on diverse antigen receptors to bind to and recognize an incredible range of potential pathogens and harmful molecules. Without the stochastic mechanisms driving this variation in expression, the proper functioning of the immune system would be severely compromised. The nervous system is similar in that it also relies on diversity in gene expression for proper functioning. Like the immune system, neurons in chemosensory systems express diverse receptors to bind a wide array of environmental molecules. In addition, neurons, even of the same class, must be sufficiently different from one another in order to properly identify self vs. non-self. Finally, pathogens also diversify their surface molecules in an arms race with the adaptive immune system. Thus, understanding the mechanisms that produce non-deterministic cellular heterogeneity is an important area of study.

In this review, we will focus specifically on non-deterministic processes that select one or a few surface molecules to be expressed on a particular cell from among many copies of similar sequences encoded in the genome. We highlight 5 such systems: the expression of variable surface glycoproteins (VSGs) by the parasite *Trypanosoma brucei*, pathogen identification by B cell and T cell receptors, neuronal self-avoidance through expression of Dscams and protocadherins, and the perception of stimuli through the olfactory system. While other reviews have compared subsets of these systems, here we broaden the scope of the comparison by considering both single-celled organisms and animals and by considering both neuronal and barrier functions (Magklara and Lomvardas 2013; Khamlichi and Feil 2018; Aresta-Branco et al., 2019a). In addition to comparing molecular mechanisms, we highlight the distinct types of utility gained by non-deterministic expression in different systems. Often, procedural or algorithmic mechanisms are simply more concise than deterministic mechanisms. In other cases, unpredictability in molecular outcomes is itself crucial for cellular function.

There are many similarities across these five examples (summarized in Table 1). First, they all have some type of restriction mechanism, often heterochromatin-based, that ensures that all of the coding sequences that could possibly be expressed aren’t expressed at the same time. Each system also involves stochastic selection of a single (or a few) isoform(s) that will be expressed. For antigen receptors, Dscams, protocadherins, and olfactory receptors, stochastic selection involves a unique enhancer or locus control region. Such a region or enhancer has not yet been identified for *VSG*s. Lastly, in three of these systems, there are feedback mechanisms downstream of selection that can act to help correct any flaws that were made during selection. In antigenic (VSG) variation, this feedback is whether or not the cell survives the host immune system. In V(D)J recombination, feedback takes place within the germinal center when higher affinity B cells win the competition for antigen. In olfactory receptor choice, the feedback mechanism allows the cell to choose a different olfactory receptor gene if it initially chose a flawed one—or stops the cell from choosing another gene if the one it already chose is functional. Although similar feedback processes may take place in Dscam and protocadherin expression, they have not yet been discovered.

**TABLE 1:** Comparison of non-deterministic systems of cell surface molecule expression. For brevity, references are not included; they are provided throughout the main text description of each system.

	VSG	IgG	Dscam	Pcdh	ORs
Combinatorial diversification	Yes—construction of mosaic VSGs increases repertoire	Yes—V, D, and J exons are variably combined	Yes—exons 4, 6, and 9 are variably combined; multiple isoforms per cell	Yes—cells can express isoforms from A, B, and G clusters; some cells express multiple genes from one cluster	No
Monoallelic	N/A—active VSG copied from a “genomic archive; ” expression sites can be hemizygous	Yes	No	Sometimes	Yes
Exclusive (i.e. exactly one isoform/cell)	Yes	Yes	No	Cells generally express isoforms from A, B, and G clusters. The choice within cluster is not necessarily exclusive	Yes
Dependence on limiting enhancer	Active VSG associates with genomic locus encoding splice-leader RNA	Yes, for proximal V promoter selection	At the RNA level, dependent on unique RNA “chooser” elements	Yes, e.g. HS5-1 for PCDHA	Yes, Greek Islands
Mechanism of choice/variation	Recombination into active site, active site switching, construction of mosaic VSGs	Recombination and AID-induced point mutation	Alternative splicing	Promoter choice via limiting enhancer(s)	Promoter choice via limiting enhancers
Expression choice in each cell is initially more promiscuous, and then refines	Yes	No	N/A, not exclusive	Yes	Yes
Choice is stable once refined	Choice is heritable. Switching is critical, but unclear how it is induced	Yes	No	Unknown	Yes
Feedback	Selection by immune system clearance	Unfolded protein response	Unknown	Unknown	Unfolded protein response
Function of non-deterministic choice	Immune system can’t predict what antigen will be expressed next, mosaic VSGs expand repertoire	Pathogen can’t predict what antibodies will be present, allows defense against novel pathogens that were not predicted by evolution	Allows neurons of the same ontogenetic type to have distinct barcodes, and allows neurons to respond differently to themselves than to ontogenetically identical sisters	Allows neurons of the same ontogenetic type to have distinct barcodes, and allows neurons to respond differently to themselves than to ontogenetically identical sisters	Provides a concise mechanism for activating OR expression; new ORs can be expressed without evolution of new transcriptional mechanism; only need one regulatory system instead of 1,000
Drawbacks of non-deterministic choice	Not obvious	Since antibodies are produced randomly, many arise that bind self-antigens. These must be selected against	Not obvious	Not obvious	Requires receptor-dependent mechanisms to wire OSNs to olfactory bulb glomeruli
Function of restricted expression and diverse cell surface phenotypes	Prolongs infection by allowing host immune system to “see” only one VSG at once	Allows binding of diverse and novel antigens; compartmentalization allows cellular somatic selection of effective receptors	Neuronal self-identification and self-avoidance	Neuronal self-identification and self-avoidance	Olfactory perception—each cell senses limited and distinct odorants

While we restrict our analysis here to mechanisms that diversify cell surface molecule repertoires by choosing among genetically encoded paralogues, we note that all biological diversification ultimately relies on noise in genome replication that produces mutations, and that noise is often harnessed and regulated to do biological work. For example, HIV immune evasion has been suggested to result from the virus’s retention of an unusually error-prone replication enzyme, and switches between lytic and latent phases are thought to occur stochastically (Roberts et al., 1988; Weinberger and Weinberger 2013; Cuevas et al., 2015). Behavioral switches are also likely governed by probabilistic rather than deterministic mechanisms. The degree of variation in gene expression between cells is itself subject to selection, and such variation can alter the penetrance of mutant alleles (Raj et al., 2010; Metzger et al., 2015; Duveau et al., 2018) Stochastic processes can also reduce the fitness costs of mutations, as in the case of X inactivation in female mammals. While the processes of life contravene entropy, in many cases the otherwise robust and predictable mechanisms of cellular development allow molecular noise to peek through in a regulated manner to influence phenotype.

As we discuss throughout, the monogenic and/or monoallelic expression of cell surface molecules allow each of these systems to appropriately interact with the outside or extracellular world. The functional purpose of selecting cell surface molecules in a non-deterministic rather than a predictable manner varies across them. In some cases, non-deterministic processes may be the only way for cells in otherwise almost identical environments and with identical differentiation regimens to become distinct from one another. In the nervous system, for example, groups of neurons that are developmentally equivalent and located in the same location can produce different cell-surface proteins by randomly selecting and expressing certain gene segments or genes—as is the case with olfactory receptors in olfactory sensory neurons. Non-deterministic expression systems likely also allow for a larger array of different proteins to be made than can otherwise be deterministically encoded by the genome, as is likely the case for the immune system. Because it is inherently unpredictable, non-deterministic expression may also increase fitness for hosts and pathogens locked in battle with one another.

### Definitions

In these fields, the terms “stochastic” and “random” are used to refer to processes in which knowing the ontogenetic identity of a cell predicts a distribution of possible gene expression choice but is insufficient to deterministically predict cell surface molecule expression. We note that in most of these systems, molecular choices follow biased distributions—for example, olfactory receptor choice is biased by position in the olfactory epithelium, VSG choice by the time course of infection, and Dscam choice by the neuronal cell type. Biased distributions are consistent with the mathematical definitions of stochastic or random, and we continue to use those terms here. We use the terms “non-deterministic,” “probabilistic,” and “unpredictable” as additional descriptors. Further, we use “monoallelic” to refer to molecular choice between two copies of the same gene, and “monogenic” to refer to selection among paralogues. We include gene families here, for example the Dscams, in which surface molecule choice is not exclusively monogenic, i.e. where multiple choices are made in each cell but most of the available choices are still repressed. We note that in the VSG field, expression of a single VSG paralogue per trypanosome is typically referred to as “monoallelic” expression. For consistency with the other topics covered here, we use the term “monogenic.”

## Variant Surface Glycoproteins

**FIGURE 1:**
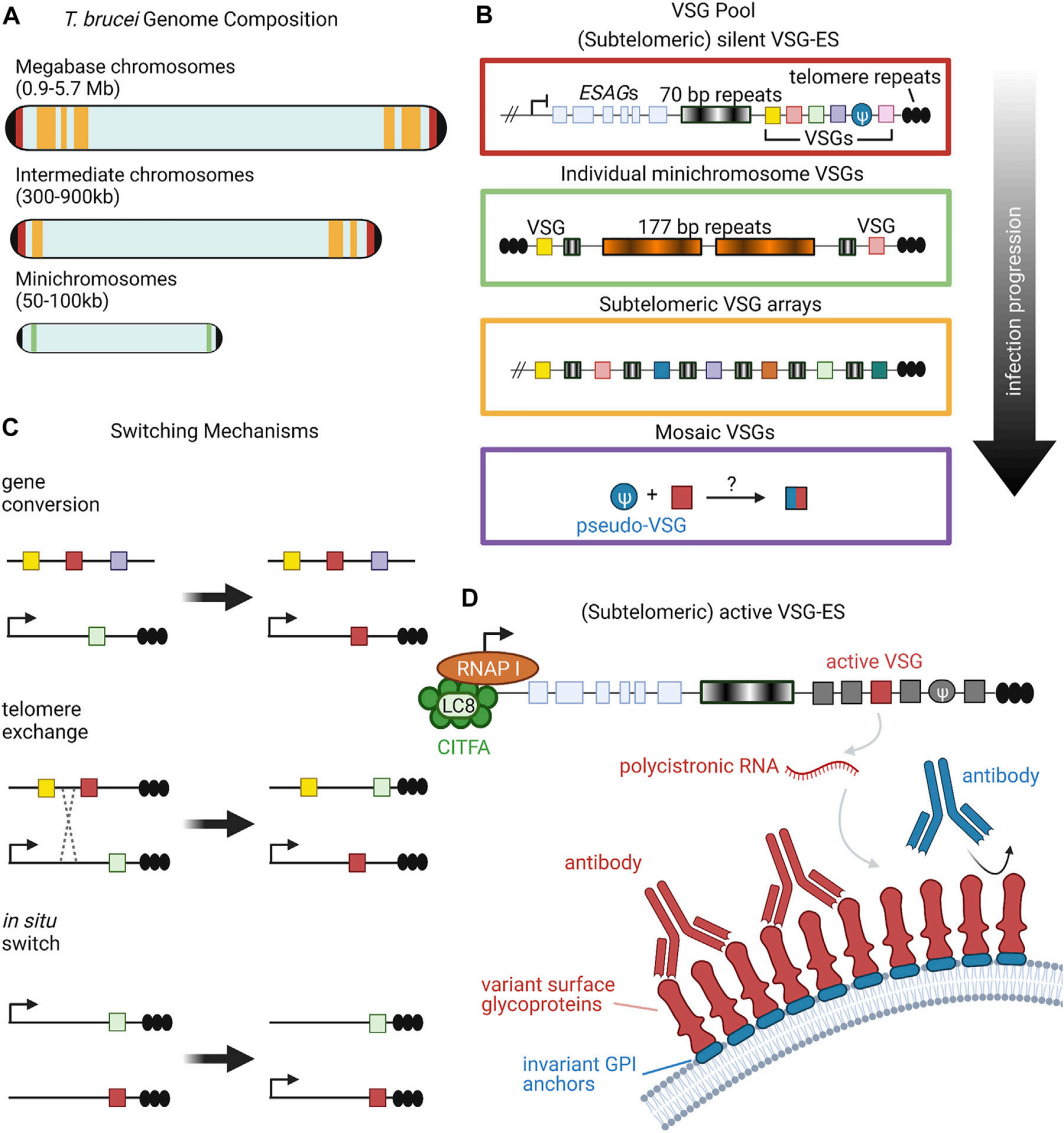
Organization and Expression of VSGs. **(A)** *T. brucei* has an unusual karyotype consisting of large, megabase-sized chromosomes, intermediate chromosomes, and minichromosomes. Size ranges of each chromosome type are listed in parentheses (Berriman et al., 2005). Black caps on the ends of the chromosomes represent telomeres. Red, yellow, and green bands denote the typical locations of different VSG repertoires, corresponding to the colored-coded insets in panel **(B)**. **(B)** Candidate VSG genes are located on megabase and intermediate chromosomes within silent subtelomeric bloodstream expression sites (VSG-ES; red box) or subtelomeric arrays (yellow box). Individual VSG genes can also be found in subtelomeric regions of minichromosomes (green box) or can be generated from recombination of intact and/or VSG pseudogenes from various sources (purple box). Throughout the course of infection, *T. brucei* will draw upon the VSG pool in a semi-predictable manner according to the location of candidate genes; for example, VSG gene arrays from silent expression sites are typically used before minichromosome VSGs (Sima et al., 2019). **(C)** VSG expression proceeds from a single active expression site. To shift expression to a new VSG gene, *T. brucei* can employ one of three switching mechanisms: gene conversion, telomere exchange, or *in situ* switching (Liu et al., 1983; Rudenko et al., 1996; Horn and Cross, 1997; Robinson et al., 1999; Li, 2015). **(D)** RNA polymerase I transcribes polycistronic RNAs from active VSG expression sites. The CITFA transcription factor complex, which in *T. brucei* consists of CITFA subunits 1–7 (green circles) complexed with LC8/DYNLL1 (light green oval), is a basal transcription factor required for RNA pol I initiation (Kirkham et al., 2016). The active VSG gene is typically transcribed last, preceded by expression-site associated genes (ESAGs) (Pays et al., 2001; Hertz-Fowler et al., 2008). VSG RNA is translated into variant surface glycoproteins, which form a densely-packed coat that prevents recognition of underlying invariant cell-surface molecules, such as GPI anchors (Hertz-Fowler et al., 2008; Horn and McCulloch, 2010). Figure inspiration was drawn from various sources (Rudenko et al., 1996; Berriman et al., 2005; Li, 2015). All figures in this review were created using BioRender.

Many pathogens have learned to survive in host environments that are hostile to their growth. One such method that pathogens have evolved is antigenic variation. Here, we will discuss coat protein switching in trypanosome infection as a model. *Trypanosoma brucei* is a single-celled eukaryotic pathogen that has dedicated a large amount of its genome to this process. This parasite is found mostly in sub-Saharan Africa and is the cause of a vector-borne disease known as sleeping sickness. *T. brucei* is coated by a dense layer of variant surface glycoproteins (VSGs) and is able to switch which *VSG* is expressed in order to evade host immune systems (Boothroyd et al., 2009; Hoeijmakers et al., 1980; Sima et al., 2019). The high density of surface VSG molecules shields the pathogen’s other non-variable surface proteins (Figure 1), making the pathogen’s immunological identity tied to the particular *VSG* it expresses (Hertz-Fowler et al., 2008; Horn and McCulloch, 2010).

Research suggests that there are about 2000 genes that constitute the *VSG* repertoire of *T. brucei,* clustered into subtelomeric arrays as well as on several minichromosomes (Figure 1A) (Cross et al., 2014). Though each individual *T. brucei* organism expresses only a single *VSG* gene at a time, VSG switching has been shown to occur at a rate as high as 10^–3^ switches per cell per generation (Mugnier et al., 2015; Turner and Barry, 1989). This high switching rate, along with the large number and diversity of cells present, leads to a sinusoidal pattern of infection where the immune system eliminates cells expressing a given VSG, but not before new variants arise in the population. These variants then grow in number, only to be wiped out again by the immune system, followed by the emergence of new variants (Mugnier et al., 2016). This constant back-and-forth between new VSG variants and the host immune system allows for *T. brucei* to remain inside of a host for long periods of time, creating chronic infections. Interestingly, studies of *T. brucei* population dynamics have begun to reveal semi-predictable patterns in *VSG* expression based on gene location and other gene family characteristics (Figure 1B), but much is still unknown about the level of determinism in the system (Morrison et al., 2005; Mugnier et al., 2015).

Multiple overlapping mechanisms accomplish this dynamic VSG switching (Figure 1C). The predominant mechanism is duplicative *VSG* gene conversion, in which a silent *VSG* gene is copied into an active expression site (Liu et al., 1983; Robinson et al., 1999; Li, 2015). *VSG* expression can also swap via *in situ* switching where a previously silenced expression site is activated, while the previously active site is silenced (Horn and Cross, 1997). A third mechanism is telomere exchange, where telomeric regions undergo crossing over that swaps which *VSG* is downstream of the active promoter (Rudenko et al., 1996). The field has primarily focused on these first two mechanisms, with telomere exchange still relatively underexplored, so we will focus more on gene conversion and *in situ* switching in this review.

As in all the monogenic expression systems described in this review, repression of the majority of possible loci is a necessary condition for restricted use of the chosen locus. The sub-telomeric location of *VSG* expression sites plays a part in their repression (Ersfeld et al., 1999; Berriman et al., 2005). Telomere proximity is inversely related to transcriptional activity of genes generally (Robin et al., 2014), and this trend holds true for DNA Pol I transcribed genes such as *VSG* genes (Glover and Horn, 2006). The telomere binding protein RAP1 is an essential component of the telomere complex and has been associated with *VSG* repression (Yang et al., 2009). The protein phosphatidylinositol 5-phosphatase binds to RAP1, and, together with phosphatidylinositol 5-kinase, helps to control *VSG* gene repression near the telomere by phosphorylating and dephosphorylating key regulatory molecules (Cestari and Stuart, 2015).

### VSG Expression Sites

The trypanosome genome has 20–40 polycistronic, sub-telomeric expression sites (ES) that promote transcription of *VSG*s as well as ES-associated genes (*ESAG*s) (Hertz-Fowler et al., 2008; Pays et al., 2001). They contain a Pol I promoter and are typically around 45 kb in length, with the *VSG* gene the most distal gene transcribed (Figure 1D) (Pays et al., 2001; Hertz-Fowler et al., 2008). We will focus our attention on expression sites active during the bloodstream stage of expression (bloodstream expression site, BES). Interestingly, though VSG proteins are monogenically expressed, it has been observed that multiple BES can be transcriptionally active at a time (Kassem et al., 2014). The additional BES transcripts do not fully elongate, are transcribed at lower levels, and are not translated, indicating additional regulation at the transcriptional and post-transcriptional levels to maintain monogenic expression (Kassem et al., 2014). Due to differing recombination into BESs, the two alleles of a particular BES could contain different contents; regardless, expression is from only one BES per cell and is therefore monoallelic.

### VSG Induction, Inheritance, and Switching

Monogenic expression and switching of a single VSG gene is what allows the parasite to successfully evade the host immune system. In fact, parasites that express multiple VSG proteins at once are quickly cleared by the immune system (Aresta-Branco et al., 2019b). VSG expression initiates in parasites that reside in the salivary gland of the tsetse fly, prior to bloodstream infection. Recent data has shown that multiple VSG genes are initially transcribed within pre-metacyclic cells, with a single gene being expressed within mature metacyclic cells (Hutchinson et al., 2021). A “race” model has been proposed to explain this phenomenon in which different VSG expression sites race to hit a certain threshold level of transcription. Once a particular gene hits this threshold, the other transcribed expression sites become downregulated, possibly due to the limited expression machinery being used up at this single site, or by the actively transcribed RNA transcripts silencing expression at the other sites (Hutchinson et al., 2021). The particular transcribed VSG and its localization to the nuclear expression site can be inherited following cell division and this inheritance depends on the chromatin assembly factor CAF1 (Faria et al., 2019). Remarkably, simply loosening chromatin structure through ectopic overexpression of the high-mobility group box protein TDP1 is sufficient to allow expression of multiple VSGs per cell (Aresta-Branco et al., 2019b).

While the choice of active VSG can be stable within the life of a cell and through cell division, occasional VSG switching is critical for immune evasion and long-term infection. How VSG switching is regulated—whether this is a probabilistic event or induced by parasite or host factors—remains unknown. The molecular mechanisms that induce VSG exchange are also mysterious. Some possible explanations include collapse of the replication fork due to continuous VSG transcription (Glover et al., 2013), or translocations triggered by frequent DNA damage, such as double stranded breaks, within unstable regions surrounding expression sites (Boothroyd et al., 2009). However, loss of RECQ2, a helicase which repairs DNA breaks within the telomere, leads to an increase in DNA recombination, indicating a possibility that double stranded breaks are not responsible for inducing VSG switching (Devlin et al., 2016). In contrast, VSG transcription and DNA replication have been shown to be associated with one another (Devlin et al., 2016). Thus, VSG switching could be induced by DNA fragility brought about by DNA replication (Devlin et al., 2016).

Telomere length, telomere stability, and the regulation of the chromatin structure surrounding VSG expression sites has also been shown to be important for VSG switching (Hovel-Miner et al., 2012; Aresta-Branco et al., 2016). The degree to which this VSG expression choice is stochastic versus deterministic has also come into question with studies revealing a degree of predictability in VSG emergence, which could result from either ordering of VSG choice or from differential selection (Morrison et al., 2005; Mugnier et al., 2015).

### Expression Site Activation and In Situ Switching

**FIGURE 2:**
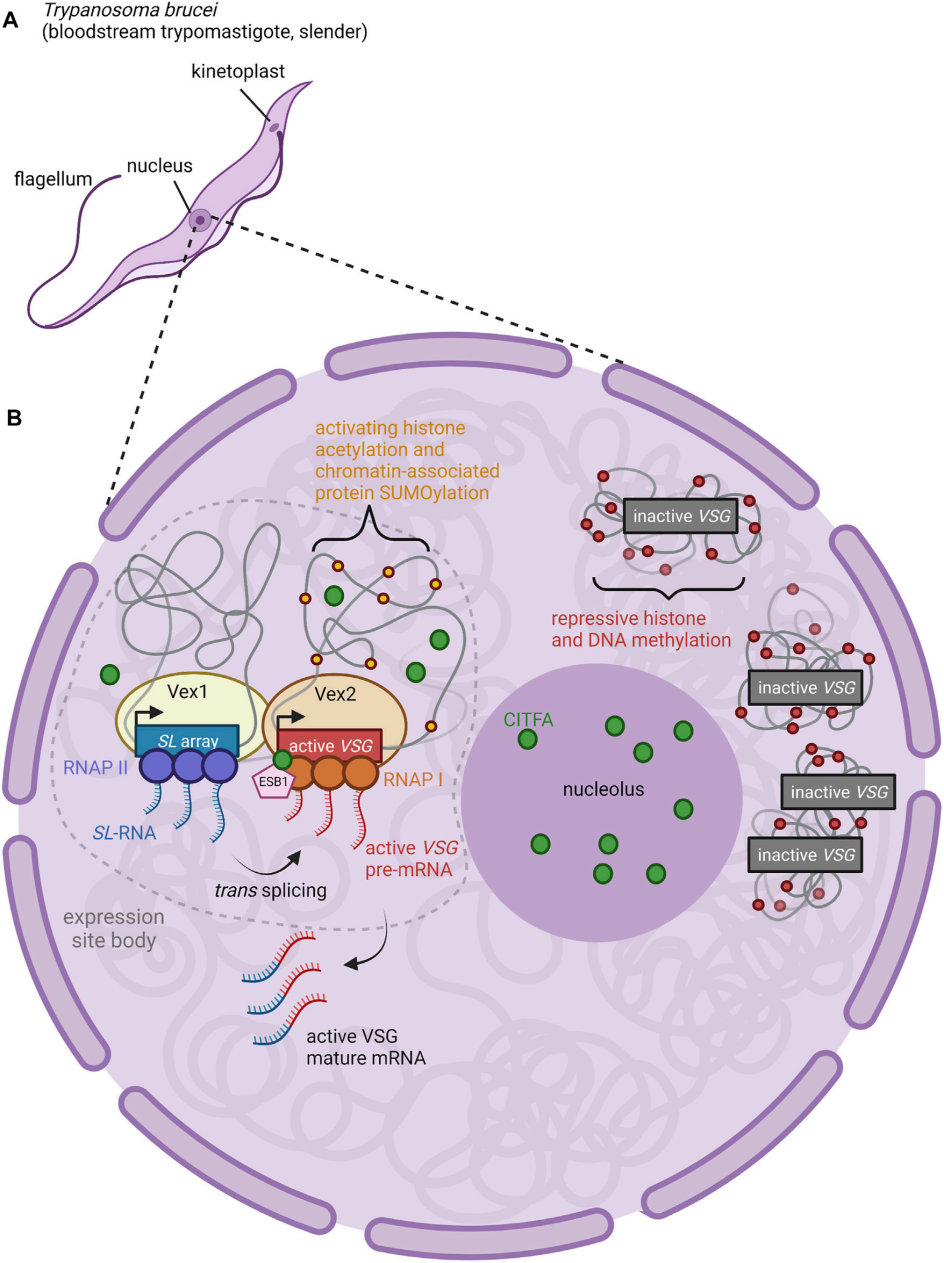
Nuclear Organization and Control of VSG Expression. **(A)** Anatomy of a *T. brucei* cell. The long slender shape is characteristic of the bloodstream form of the parasite, an actively proliferating stage which causes chronic parasitemia in infected hosts (Matthews, 2005). **(B)** The active BES VSG is expressed within an extranucleolar expression site body (ESB) (Navarro and Gull, 2001). ESBs are enriched for RNA Pol I, CITFA transcription factor complexes (green circles), and the ESB-specific transcription factor ESB1 (pink pentagon) (Brandenburg et al., 2007; Nguyen et al., 2012; Nguyen et al., 2014; Escobar et al., 2021). Both CITFA and ESB1 are required for transcription of the active VSG gene; the absence of these factors from inactive VSGs (relegated to heterochromatic regions outside of the ESB) contributes to the repression of inactive VSGs (Brandenburg et al., 2007; Nguyen et al., 2012; Nguyen et al., 2014; Escobar et al., 2021). Within the ESB, the proteins VEX1 (yellow oval) and VEX2 (light orange oval) complex together, associating an mRNA splicing locus (SL array, blue rectangle; RNAP II, purple circles) with the active VSG expression locus (active VSG gene, red rectangle; RNAP I, orange circles) (Glover et al., 2016; Faria et al., 2021). Thus, transcription of the splice leader (SL) RNA by RNAP II proceeds adjacent to transcription of the active VSG pre-mRNA by RNAP I. The 5′ end of the SL RNA is then spliced in trans to the VSG pre-mRNA to form mature VSG mRNA (Faria et al., 2021). Figure inspiration was drawn from various sources (López-Farfán et al., 2014; Martínez-Calvillo et al., 2019; Nguyen et al., 2014; Escobar et al., 2021; Faria et al., 2021).

While only one BES at a time produces an actively translated product, the active BES can switch between the repertoire of available BES through *in situ* switching (Figure 1C). Several factors have been identified as characteristic features of the active BES that must be altered in order for *in situ* switching to occur (Cestari and Stuart, 2018). Reminiscent of the importance of nuclear organization in *OR* gene selection (described below), active BES are localized to an extranucleolar region termed the expression site body (Figure 2) (Navarro and Gull, 2001). The expression site body and active BES promoter are enriched for Pol I along with the *basal class I transcription factor A* (CITFA) complex (Brandenburg et al., 2007; Nguyen et al., 2012; Nguyen et al., 2014). The novel transcription regulator NLP similarly associates selectively with the active BES (Narayanan et al., 2011).

What mechanisms ensure that only a single BES can produce functional VSG in each cell? Work in the last 5 years has focused on two factors identified in genetic screens that are required for VSG expression: *VSG exclusion 1* (*VEX1*) and *VSG exclusion 2* (*VEX2*). VEX1 has been shown to positively regulate the active VSG site in *cis* while also negatively regulating all other VSGs in *trans* (Glover et al., 2016). VEX1 binds to VEX2 independently of transcription, and together, they are responsible for VSG exclusion (Faria et al., 2019). Recent work suggests that the single VSG chosen for expression in a particular cell is physically associated with the genomic locus that encodes mRNA splice-leader sequences (Faria et al., 2021). In *T. brucei*, mature mRNA is produced by *trans*-splicing to leader sequences produced from this locus. The active BES can associate with the splice-leader locus in *trans*, across chromosomes (Faria et al., 2021). VEX1 associates with the splice leader locus, and VEX2 with the actively expressed VSG in the BES.

Thus, VSG transcription and mRNA splicing take place within a specific compartment of the nucleus and are associated closely with VEX1 and VEX2 proteins (Figure 2) (Faria et al., 2021). The coalescence of the VEX1-bound splice leader locus and the VEX2-bound BES into the expression site body may serve to activate Pol I transcription and subsequently induce repression of the remaining BESs (Glover et al., 2016; Schulz and Papavasiliou, 2016; Cestari and Stuart, 2018; Faria et al., 2021). Transcription-mediated gene silencing is a hallmark of heterochromatinization in other stochastic systems such as yeast mating-type switching, and could be involved in olfactory receptor heterochromatinization as well (Allshire and Madhani, 2018). Another possibility is that failure to concentrate access to the splice leader cassette and transcription factors on one VSG gene allows multiple VSGs to be expressed at lower levels.

Chromatin modifications also appear to play an important role in BES activation. The active BES is significantly depleted of histones, especially H3, in comparison to other, silent BESs (Stanne and Rudenko, 2010). In support of this, the knockdown of H1, H3, H3.V, and H4.V increased the accessibility and transcription at previously inactive BES promoters and *VSG* genes (Povelones et al., 2012; Reynolds et al., 2016; Schulz et al., 2016; Müller et al., 2018). This effect has been shown to be mediated by chromatin remodeling proteins such as ASF1A, CAF-1b, and SIR2rp1, alongside a handful of histone methyltransferases and acetylases/deacetylases (Alsford et al., 2007; Figueiredo et al., 2008; Kawahara et al., 2008; Wang et al., 2010; Alsford and Horn, 2012). SUMOylation also plays a crucial role, with SUMOylated chromatin-associated proteins serving as a distinct marker of the active BES in the expression site body (López-Farfán et al., 2014). The *VSG* transcriptional activator SNF2PH is recruited to SUMOylation-rich BES where it is itself SUMOylated to subsequently facilitate Pol I transcription (Saura et al., 2019). Pol I is then further regulated by activating SUMOylation via TbSIZ1/PIAS1 (López-Farfán et al., 2014). All of these SUMOylation events appear to be localized to the active BES.

While nuclear localization, transcription factor recruitment, and chromatin modification have all been shown to be relevant in *VSG* expression at the selected BES, the order and dominance of these activating events is still unclear. It is also still uncertain what induces these activation signals to switch between BESs during *in situ* switching.

### Duplicative Gene Conversion

Early genetic experiments revealed that VSG switching can involve the overwriting of genomic loci (Horn, 2014). A form of recombination, duplicative gene conversion involves the removal of the active *VSG* gene from the expression site, which is replaced by a duplicated form of a previously silent *VSG* gene (Figure 1C) (Liu et al., 1983; Robinson et al., 1999). Boothroyd et al. found that gene conversion switches are initiated by DNA double-strand breaks which are subsequently repaired by homologous recombination (Boothroyd et al., 2009). Each *VSG* gene possesses an upstream region of 70 bp repeats. Double-strand breaks adjacent to these repeats were both necessary and sufficient to induce VSG switching, suggesting that the repeats serve as a guide for homologous recombination that allows for the active *VSG* site to be overwritten (Boothroyd et al., 2009). The BESs, along with many of the silent *VSG* arrays, are located in sub-telomeric regions of the genome (Cross et al., 2014). These regions are inherently unstable portions of the genome where recombination and double-strand breaks frequently occur (Glover et al., 2013; Horn, 2014). As such, it is suspected that *T. brucei* takes advantage of this natural instability to induce *VSG* gene conversion; however, alternative mechanisms for DNA lesion production have been proposed (reviewed in da Silva et al., 2018). *VSG* recombination requires RAD51 and BRCA2, while TOPO3α has been shown to suppress recombination and restrict it to the 70bp repeats in partnership with RMI1 (McCulloch and Barry, 1999; Hartley and McCulloch, 2008; Kim and Cross, 2010; Kim and Cross, 2011).

### Mosaic VSGs

While trypanosomes predominantly switch between existing intact *VSG* genes at the beginning of an infection, long read sequencing has confirmed that there is a significant increase in the number of novel mosaic *VSG* genes as infection time increases (Jayaraman et al., 2019; Mugnier et al., 2015). Because 80% of *T. brucei’s* ∼ 2000 *VSG* genes are incomplete or pseudogenes, the repertoire of complete genes is eventually exhausted during chronic infections (Berriman et al., 2005; Cross et al., 2014). Once the majority of complete genes have been expressed and recognized by the immune system, *T. brucei* utilizes segmental gene conversion to merge fragments of different *VSG* genes in what is termed mosaic recombination (Figure 1B) (Mugnier at el., 2015). This process is not uncommon, as other pathogens have been known to utilize segmental gene conversion to further diversify their pool of variant genes (Zhuang et al., 2007). Trypanosomes are able to construct functional mosaic *VSG* genes from pseudogenes and gene fragments, suggesting that *T. brucei’s* large abundance of partial genes are important for continued diversification (Hall et al., 2013). It is still unclear what cellular process is used to merge the *VSG* segments together. One possibility is that mosaics are generated by homologous recombination within the *VSG* gene similar to duplicative recombination or by crossover events, as in telomeric exchange. It is also unknown whether mosaic formation occurs within expression sites, or if instead they are formed somewhere else in the genome before being moved into the expression site.

Sleeping sickness remains a deadly and difficult to treat disease, so increasing our understanding of the mechanisms that allow this parasite to evade host immune systems will provide advances in our ability to fight *T. brucei* infections. A more detailed analysis of remaining questions in the field is reviewed by McCulloch and colleagues (McCulloch et al., 2017). Similar methods of variation utilized by trypanosomes can also be found in the systems they are meant to evade. Just as antigen diversity aids pathogens in evading the immune system, antigen receptor diversity allows for greater detection. B and T cells in the immune system create this diversity through stochastic genome editing. This process is often initiated by the introduction and subsequent repair of DNA double-stranded breaks, similar to gene conversion in *VSG*’s (Papavasiliou and Schatz, 2000). It has also been suggested that B and T cells expand their receptor diversity via segmental gene conversion, similar to mosaic *VSG*s (Barbet and Kamper, 1993).

## Antigen Receptor Diversity

The coexistence of host and pathogen has largely driven the diversification of both the host’s immune surveillance and the pathogen’s antigenic determinants (Chang et al., 2011). Mammalian genomes contain approximately 20,000 protein-coding genes, and yet the B and T cells of the adaptive immune system produce receptors that can bind to a vast array of arbitrary antigens regardless of evolutionary experience. Receptor-level diversity was ultimately shown to be produced via two stochastic processes: V(D)J recombination, which alone can generate 10^11^ possible binding domains, and somatic hypermutation, which can introduce mutations in any of these recombination products to further expand receptor possibilities (Janeway et al., 2001). These processes allow for a truly outstanding level of diversity to emerge from just a few germline-level genes, preparing the immune system for any antigen it might face without taking up very much genomic space. Much as learning mechanisms in the nervous system allow animals to relate arbitrary sensory stimuli to the contexts in which they are experienced, selective processes during B and T cell development in the context of an immune response shape cellular immune responses according to the “meaning” of self, benign, or pernicious antigens.

Antibodies, which are immunoglobulin proteins, possess variable binding surfaces that can recognize diverse antigens. These antibodies can be secreted in the serum or bound to the surface of B lymphocytes to form B cell receptors (BCRs). T lymphocytes also have surface receptors (TCRs) that recognize antigens in combination with antigen presenting major histocompatibility complex (MHC) proteins. Like TCRs and BCRs, MHC proteins are present in the population as diverse alleles. While TCR and BCR diversity is generated via somatic mechanisms, population-level MHC diversity is maintained at the germline level via balancing selection.

### Immunoglobulin Gene Loci and V(D)J Recombination

Immunoglobulins are composed of covalently-linked heavy and light chains, both of which possess a variable N-terminus that recognizes antigens and a constant C-terminus that can recruit effectors (Schroeder and Cavacini, 2010). Here, we will focus on the generation of BCRs and antibodies as a model for immunoglobulin diversification. The germline-encoded heavy chain locus produces IgM and IgD constant regions via alternative splicing; both IgM and IgD can be membrane-bound or secreted as antibodies. DNA rearrangements of the heavy chain locus during the course of the lymphatic germinal center reaction can also produce secreted IgG, IgA, and IgE antibodies by joining the variable N-terminus to different constant regions; we will focus on variable region diversification. V(D)J recombination during lymphocyte development produces the initial diversity of mature IgM and IgD. Once B cells bind their given antigen, somatic hypermutation in the germinal centers allows for further diversification of the variable region alongside the production of IgG, IgA, and IgE through class switching.

**FIGURE 3:**
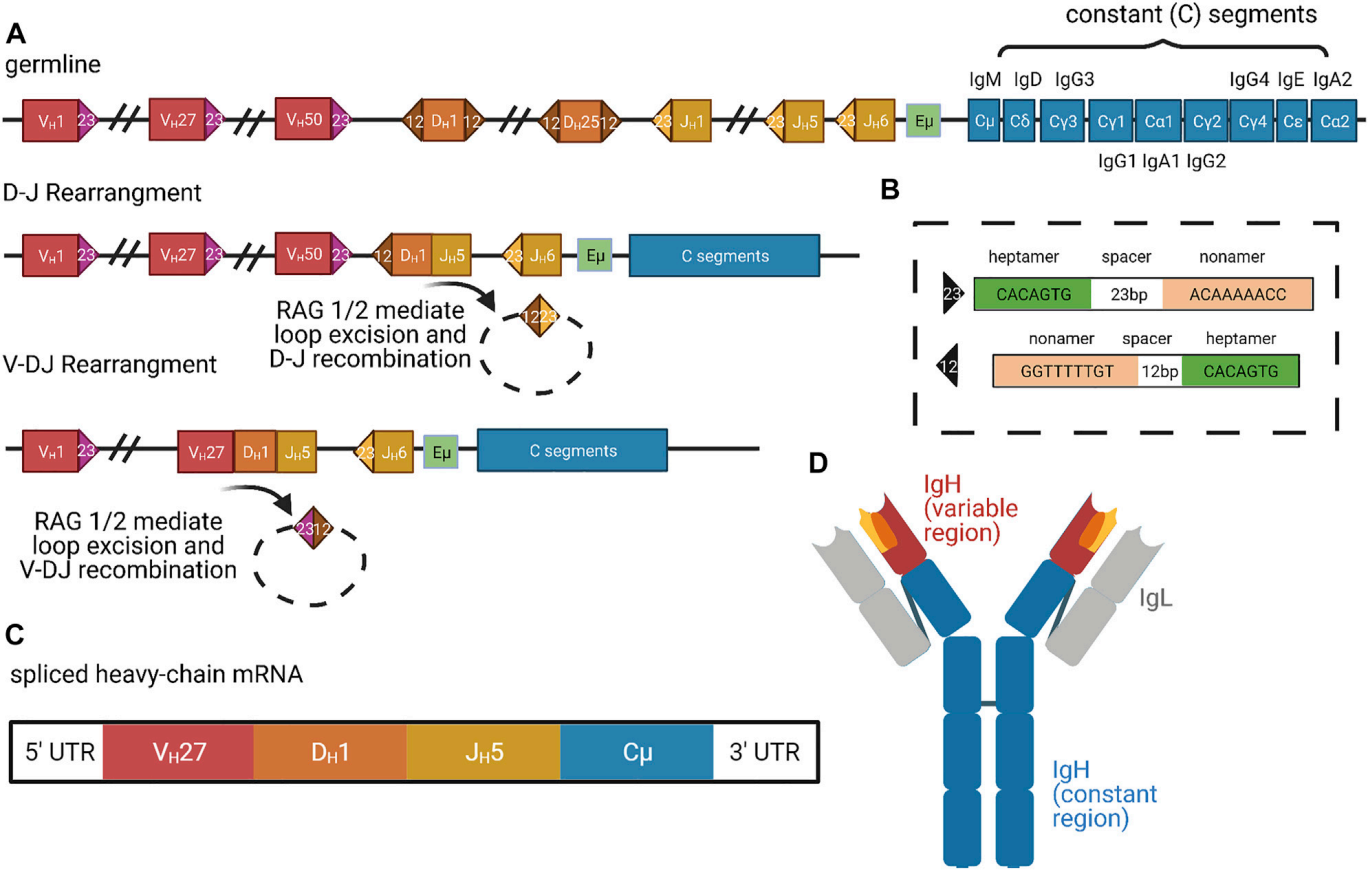
Organization and Expression of Immunoglobulins. **(A)** Successive organization of the human heavy-chain locus in the germline, after D-J rearrangement, and after V-DJ rearrangement. The germline locus contains roughly 50 V segments (red), 25 D segments (orange), 6 J segments (yellow), and 9 constant segments (blue) (Rodriguez et al., 2020). In the first step of D-J and V-DJ rearrangement, RAG1 and RAG2 complexes bind RSS motifs (colored triangles) (Schatz et al., 1989; Oettinger et al., 1990). **(B)** The RSS motifs consist of consensus heptamer and nonamer sequences, which are separated by 23 or 12 bp spacers (Ferrier, 2009). **(C)** In the rearranged DNA locus (last line of panel **(A)**), transcription only proceeds from the promoter of the most-proximal V segment because it competes most effectively for access to the limiting enhancer Eμ and other enhancers (green in **(A)**) (Roy et al., 2011). Additionally, intervening J segments are spliced out and alternative splicing selects either Cμ or Cδ constant segments for inclusion, generating IgM or IgD class BCRs, respectively. Upon activation, B cells can switch expression to different downstream C_H_ genes via additional recombination, called class switching. Thus, mature heavy-chain mRNA consists of just 1 V, D, J, and C exon (Charles A Janeway et al., 2001). **(D)** Mature IgM protein, color-coded according to contributing heavy-chain exons. Gray regions correspond to light-chain segments, which are combined with heavy chains post-translationally. Figure inspiration was drawn from various sources (Little et al., 2015; Feederle and Schepers, 2017; Backhaus, 2018).

In V(D)J recombination, the N termini of the heavy and light chains are rearranged to bring distinct V (variable) segments, followed by the D (diversity) segments, and then the J (joining) segments into proximity with the constant region (Figure 3) (Schroeder and Cavacini, 2010). The human heavy chain locus on chromosome 14 possesses roughly 50 functional V segments, 27 functional D segments, and six functional J segments (Rodriguez et al., 2020). The two major classes of light chains are kappa and lambda, both of which do not possess D segments but still undergo VJ recombination. The kappa locus is on chromosome 2 with roughly 44 functional V segments and 5 J segments, whereas the lambda locus is on chromosome 22 with roughly 37 functional V segments and only 1 J segment (Collins and Watson, 2018; Watson et al., 2015). The ability to create combinatorial V(D)J regions allows for an incredible diversity of heavy and light chains, which are both combined to further expand the possibilities for mature immunoglobulin proteins. There are roughly 3.5 × 10^6^ potential combinatorial products that can arise from these V(D)J and heavy-light chain pairings, and final protein products are additionally varied by junctional diversification that occurs during recombination. Recombination is induced by RAG1/2 (recombination-activating gene) which target discrete locations within the immunoglobulin loci through conserved and repeated DNA sequence elements (Figure 3A) (Schatz et al., 1989; Oettinger et al., 1990). As the V(D)J recombination process is “settled science,” we refer readers to other reviews or textbooks for more detailed description.

Remarkably, diversification of antigen binding repertoires through alteration of germline DNA has evolved more than once. In the lamprey, leucine rich repeat (LRR) proteins are diversified during lymphocyte maturation via insertion of LRR modules from flanking regions of the locus (reviewed in Boehm et al., 2012). Random combinatorial usage of immunoglobulin modules has also arisen in other systems: neuronal self-recognition in insects is mediated by randomized alternative splicing of the *Dscam* immunoglobulin locus.

### Enhancer-Mediated Restriction

While V(D)J recombination removes V regions proximal to D or J segments, distal V options remain intact, and each V has its own upstream promoter. Nevertheless, transcription always begins at the V region most proximal to D/J and therefore ensures that only a single V—the most proximal—is included in the transcript (Roy et al., 2011). This selection was initially thought to be performed by a limiting enhancer element located between the V and D/J domains, called Eμ (Figure 3A) (Serwe and Sablitzky, 1993; Li and Eckhardt, 2009). More recent work has suggested that several additional enhancers, including 3′RR and DICE, participate in a complex promoter selection process (Bébin et al., 2010; Roy et al., 2011). The most proximal V region promoter that remains after recombination is thought to compete most effectively for looping interactions with the limiting enhancers, thus conferring deterministic use of the most proximal V in the context of stochastic removal of alternate distal Vs. As we will describe below, the process of clustered protocadherin transcription in mammalian neuronal self-recognition also involves competition among nearly identical promoters for access to a single enhancer, and expression variability is produced by suppression of spatial bias for the proximal promoter, rather than recombination of different segment choices into proximity with the enhancer.

### Monoallelic Expression

Similar to the selective expression of a single allele in the OR, PCDH, and VSG systems, there is extensive evidence that each B cell expresses only a single BCR, which makes each B cell specific for one particular antigen (Weiler, 1965; Vettermann and Schlissel, 2010). This specificity is important for subsequent clonal selection of antibody-producing cells and proper immune response. Interestingly, the *Ig* alleles are transcribed biallelically early in B cell development, indicating that transcriptional activation alone does not govern the allelic exclusion of these loci (Singh et al., 2003). At the level of translation, transcripts from *Ig* genes that have not undergone proper V(D)J recombination possess premature stop codons that prevent production of functional protein (Bühler et al., 2004; Eberle et al., 2009). Moreover, B cells co-opt the unfolded protein response to trigger differentiation in response to BCR translation (Hetz et al., 2020). A similar process links olfactory receptor choice, stabilization of singular olfactory receptor translation, and olfactory neuron differentiation (Dalton et al., 2013).

Studies have suggested that complete V(D)J recombination of one allele induces the suppression of the second non-recombined allele, preventing subsequent recombination and productive transcription (Vettermann and Schlissel, 2010). This is supported by the observation that D-J recombination occurs in both IgH alleles, yet only one productive V-DJ recombination proceeds (Jung et al., 2006). To accomplish this, the active recombination of the locus appears to induce RAG- and ATM-mediated repositioning of the inactive allele to repressive heterochromatin alongside inducing locus decontraction that has been associated with recombination inhibition (Goldmit et al., 2005; Hewitt et al., 2009). Furthermore, the production of a complete immunoglobulin protein chain then induces progression of B cell development that subsequently downregulates RAG proteins to prevent further recombination (Grawunder et al., 1995; Galler et al., 2004). This model allows for developing B cells to make multiple attempts at performing proper recombination, as complete suppression of the alternative allele does not occur until one of the alleles has produced protein. However, in order for this process to produce a monoallelic product the induction of recombination must be asynchronous.

Early models suggested that the low rate of recombination allowed for a probabilistic first-come, first-serve mechanism where allelic selection was purely based on which allele happened to recombine first (Perry et al., 1980; Liang et al., 2004), but continued studies have revealed that the process is likely more controlled than this. It has been shown that the selected allele is replicated first and localized to the euchromatic nuclear center whereas the non-selected allele is found in the repressive heterochromatin of the nuclear periphery (Mostoslavsky et al., 2001; Skok et al., 2001). The active allele is subsequently found to have activation signatures: hypomethylation of CpG dinucleotides; hypersensitivity to DNA nucleases and restriction enzymes; and increased activating histone marks, including histone H3/H4 acetylation (Outters et al., 2015). These differences lead the two alleles to be differentially available for RAG binding and recombination (Vettermann and Schlissel, 2010). The order and significance of these influences is still unclear, and the initial mechanism that dictates the selected allele remains debated. A detailed discussion of competing models can be found here (Vettermann and Schlissel, 2010; Outters et al., 2015).

### Positive Selection of B Lymphocytes

The vast pool of antigen receptors allows for modest binding and subsequent detection of most antigens, but once lymphocytes are activated they undergo numerous rounds of selection to increase their affinity for the antigen. We will focus on the positive selection of antigen-selective B lymphocytes in the germinal center of lymph nodes. Of course, stochastic production of TCRs and BCRs can also lead to dangerous autoimmune reactions; these are minimized due to distinct processes of negative selection that occur during lymphocyte development (reviewed in Klein et al., 2014; Nemazee, 2017; Rose, 2017).

Somatic hypermutation (SHM), a key process in affinity maturation, functions to diversify BCRs and promote the adaptive immune response. During SHM, the *BCR* locus undergoes a significant increase in the rate of point mutations compared to the rest of the genome (Forrest and Oprea, 1999). These mutation “hotspots” usually encode the complementarity-determining regions in the variable N-terminus of the antibody that interact with and recognize the antigen. SHM occurs when the enzyme activation induced deaminase (AID) targets mature rearranged V(D)J and switch regions of *Ig* genes (Pilzecker and Jacobs, 2019). AID functions by binding to single-stranded DNA and removing the amino group from cytosine, which produces highly mutagenic deoxy-uracil in the DNA of both *Ig* strands at a high rate. DNA damage response processes then generate base substitutions at and around the lesion created by the deoxy-uracil (Pilzecker and Jacobs, 2019).

Lymph node germinal centers (GCs) are the site of B lymphocyte clonal selection that drives affinity maturation to produce memory B cells and antibody-secreting plasma cells (Victora and Mesin, 2014). The GC is separated into a dark and light zone. B cells undergo SHM while proliferating in the dark zone (McKean et al., 1984; Victora and Mesin, 2014). This generates a diverse clonal pool that migrates to the light zone for selection. In the light zone, B cells use their antigen receptors to retrieve antigen from the surface of follicular dendritic cells (FDCs) and then present this antigen to T follicular helper (Tfh) cells to receive survival signals. Tfh cells were found to be the limiting factor in GC selection, as they can only interact with a small portion of the B cells (Victora et al., 2010). This creates competition between B cell clones to retrieve antigen from FDCs and present it to Tfh cells, with higher affinity BCRs being able to present more antigen and receive the limited Tfh support (Victora et al., 2010). Tfh cells send support signals in the form of cytokines and cell surface receptors like CD40L, IL-21, and IL-4 to allow B cell survival and migration into the dark zone for further proliferation and SHM (Crotty, 2014). Post-transcriptional regulation of the chemokine CXCL12 receptor CXCR4, along with differential expression of polycomb proteins, have been shown to mediate zonal migration and polarization (Allen et al., 2004; Okada et al., 2005; Allen et al., 2007a; Allen et al., 2007b; Victora et al., 2010).

Multiple rounds of this selective process produces a robust pool of antibodies that have significantly improved affinity for the antigen. Though selection of B cells in the GC starts out from mostly *inter*clonal competition, competition eventually progresses to *intra*clonal competition between variants generated by SHM (Jacob et al., 1993). Some GCs will show clonal dominance of high affinity lineages, but this dominance is not required for high affinity clones to emerge and is not present in all GCs (Tas et al., 2016).

## Down Syndrome Cell Adhesion Molecules

Neural circuit wiring is an extremely important process that is highly dependent on the proper patterning of neurons within the developing nervous system. While neurons positively select their partners through recognition of deterministically expressed cell surface molecules, neurons also have to avoid synapsing with themselves in order to establish their typical anatomies and heterologous partners. This process, called neuronal self-avoidance, requires neurons to distinguish “self” from “nonself.”

**FIGURE 4:**
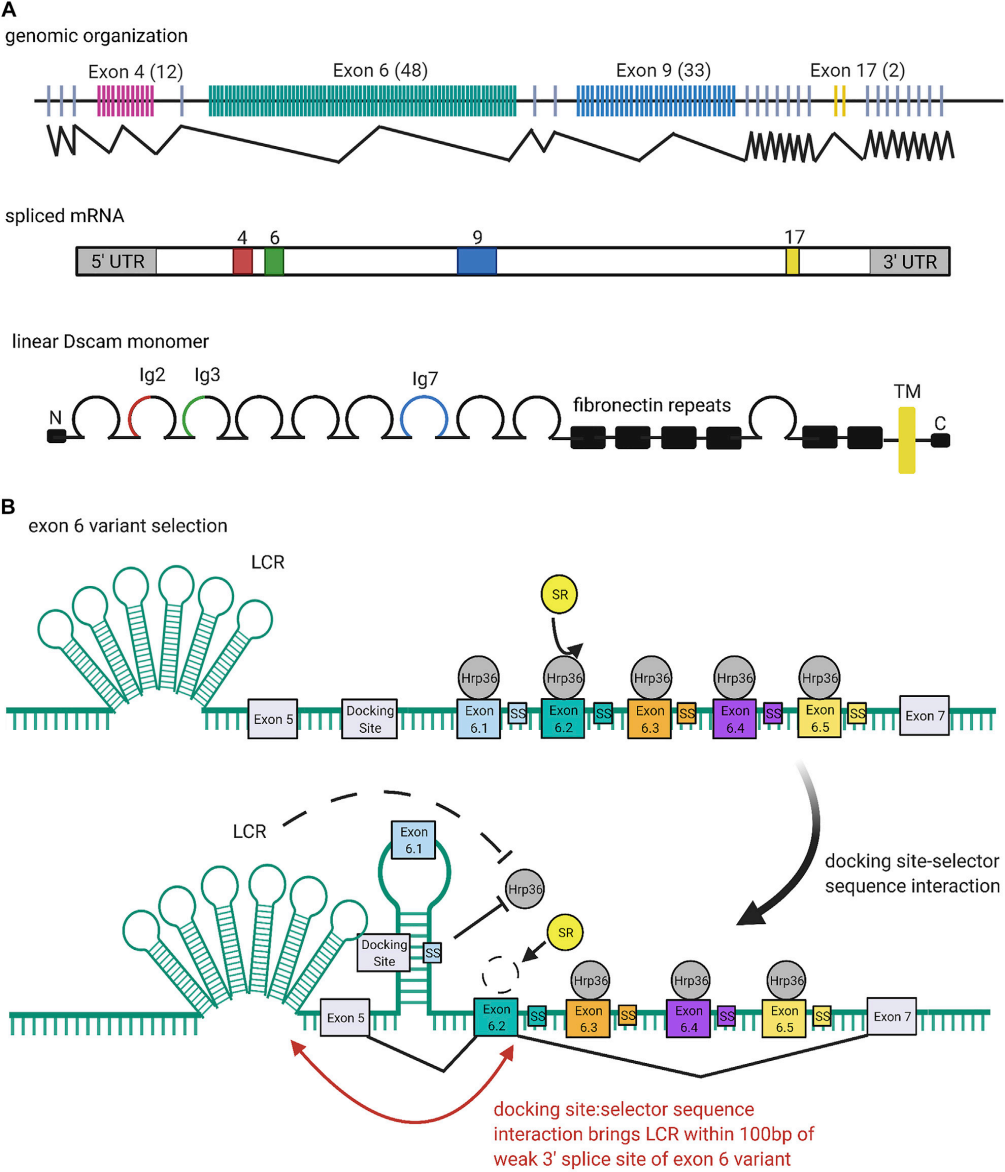
Organization and Expression of Dscams. **(A)** Genomic organization of the *Dscam1* locus in *D. melanogaster*. Numbers of exon variants are listed in parentheses next to the exon cluster number. Alternative splicing at exon clusters 4, 6, 9, and 17 (black zigzagging lines) proceeds independently, such that the *Dscam1* locus can combinatorially encode up to 38,016 unique isoforms (Schmucker et al., 2000). Exon 4 (red) and exon 6 (green) variants encode the N-terminal halves of the Ig2 and Ig3 domains, respectively, while exon 9 (blue) variants encode the entire Ig7 domain (Schmucker et al., 2000; Zhan et al., 2004). Exon 17 (yellow) codes for one of two alternative transmembrane domains, which appear to play a role in the subcellular localization of Dscams (Wang J. et al., 2004; Yang et al., 2012; Liu et al., 2020). **(B)** Mechanism of exon six variant exon selection; for simplicity, only the first 5 exon six variants and selector sequences (colored boxes) are shown, flanked by exons 5 and 7 and the common upstream docking site (grey rectangles). The exon 6 cluster of the *Dscam1* locus is maintained in a globally repressed state by binding of Hrp36 proteins (dark grey circles) to each variant (Olson et al., 2007). Binding of a variant selector sequence to the docking site forms an RNA hairpin loop which prevents inclusion of variants contained within the loop but promotes inclusion of the variant immediately downstream (Graveley, 2005; Anastassiou et al., 2006; May et al., 2011; Hemani and Soller, 2012; Xu et al., 2019). This interaction also brings the locus control region (LCR) within 100 bp of the weak 3′ splice site of the downstream variant, which may also promote variant inclusion by facilitating recognition of the splice site (Wang X. et al., 2012). Both the docking-site selector sequence interaction and LCR are thought to promote variant inclusion by antagonizing binding of the repressive Hrp36 proteins, which allows binding of inclusion-promoting SR proteins (yellow circles) at the selected variant (Olson et al., 2007; Wang S.-Z. et al., 2012). Figure inspiration was drawn from various sources (Schmucker et al., 2000; Wang X. et al., 2012).

In both vertebrates and insects, neuronal self identity is determined by randomized expression of subsets of possible cell surface molecules. These expression patterns are distinct across individual neurons, even neurons of the same type, and can be thought of as a unique barcode displayed on the surface of each individual cell. In *Drosophila*, the protein family used for this purpose is the Dscam (Down syndrome cell adhesion molecule) family of immunoglobulins. Via alternative splicing, the *Drosophila Dscam1* locus encodes up to 38,016 distinct Dscam isoforms, all of which contain the same basic structure: an ectodomain comprised of 10 immunoglobulin (Ig) domains and six fibronectin type III repeats, a transmembrane domain, and a C-terminal cytoplasmic tail (Figure 4A) (Schmucker et al., 2000). Four variable domains are encoded by clusters of exon variants, which are spliced independently of each other: *Ig2* (12 variants), *Ig3* (48 variants), *Ig7* (33 variants), and the transmembrane domains (2 variants). This means that for 38,016 distinct isoforms there are potentially (12 × 48 × 33 = 19,008) distinct ectodomains; at least 18,048 of these ectodomains are confirmed to support “homophilic” binding, or binding between identical isoforms (Wojtowicz et al., 2007).

Homophilic binding of two Dscams generates a repulsive response. When coupled with the immense diversity of Dscam isoforms, which makes it unlikely that neighboring neurons will express identical sets of isoforms, it becomes clear how Dscams mediate neuronal self-avoidance: neurites within the same neurons will express the same Dscams and repel each other, while neurites between neighboring neurons will express different Dscams and allow synapsing. The power of this “barcoding system” is evident from mutation and ablation studies: Where *Dscam1* function is disrupted, dramatic defects in self-recognition are observed, including increases in intraneuronal dendritic crossing in dendritic arborization (da) neurons (Hughes et al., 2007; Matthews et al., 2007; Soba et al., 2007) and failure of sister branch segregation in the axons of mushroom body (MB) neurons (Wang et al., 2002; Hattori et al., 2007). In addition to self-recognition, Dscams have also been suggested to mediate synaptic target selection and axon guidance in several kinds of neurons (Wang et al., 2002; Hummel et al., 2003; Zhan et al., 2004; Zhu et al., 2006; Millard et al., 2010).

### Structure and Function of Dscam Homophilic Binding

**FIGURE 5:**
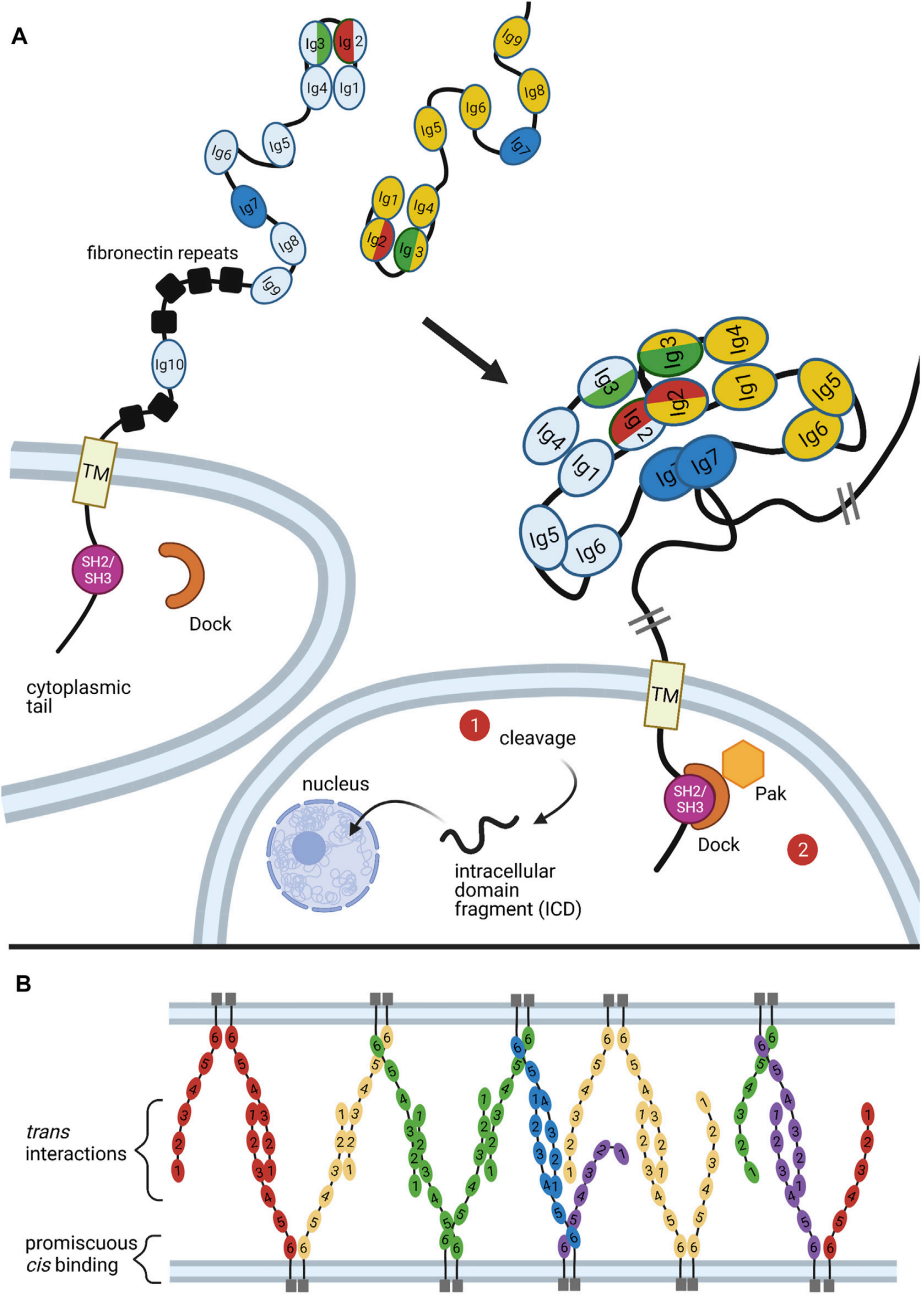
Binding of Dscams and Protocadherins. **(A)** Homophilic binding of Dscam domains Ig1-Ig8 produces an S-shaped dimer (Meijers et al., 2007; Sawaya et al., 2008). The variant domains Ig2, Ig3, and Ig7 confer binding specificity, with the variant halves of Ig2 (red) and Ig3 (green) forming a composite binding interface and Ig7 (blue) binding independently (Sawaya et al., 2008). Upon homophilic binding, a repulsive signal is generated (Matthews et al., 2007). While it is still unclear what mediates this signal, two possible pathways are illustrated. First, studies in mammalian DSCAMs revealed a nuclear localization signal (NLS) in the cytoplasmic tail. Upon cleavage of the tail, the NLS promotes translocation of the tail fragment into the nucleus, where it affects expression of synapsing genes. Although similar cleavage and nuclear translocation has not yet been demonstrated in flies, fly Dscams do have a conserved NLS in the cytoplasmic tail (Sachse et al., 2019). Second, the adaptor protein Dock has been shown to bind the SH2/SH3 domains of the Dscam cytoplasmic tail and recruit the effector kinase Pak1 (Hing et al., 1999). But while Pak1 has been shown to mediate axon guidance, it does not seem to be necessary for neuronal self-avoidance (Hughes et al., 2007). **(B)** Similar to Dscams, protocadherins bind homophilically. A mixture of *trans* and *cis* interactions forms a zipper-like lattice spanning neighboring cell membranes (Brasch et al., 2019). Figure inspiration was drawn from various sources (Sawaya et al., 2008; Schmucker and Chen, 2009; Guo et al., 2012; Chen and Maniatis, 2013; Goodman et al., 2017).

As discussed, binding specificity is critical to Dscams function in neuronal self-avoidance (Neves et al., 2004; Zipursky et al., 2006). Indeed, both *in vitro* and *in vivo* studies have demonstrated highly specific homophilic binding, to the point that isoforms differing in just a few residues exhibit very weak or no binding (Wojtowicz et al., 2004; Zipursky and Sanes, 2010). How is this exquisite specificity determined? Furthermore, how does attractive homophilic binding generate a repulsive response? Briefly, ELISA binding assays have determined that the 8 N-terminal domains (Ig1-Ig8) of Dscam proteins are sufficient to support normal binding (Figure 5A) (Wojtowicz et al., 2004). Contained in this sequence are the Ig2, Ig3, and Ig7 variant domains, which determine the binding specificity of isoforms by selectively “matching” with their identical counterparts (Wojtowicz et al., 2004; Wojtowicz et al., 2007; Sawaya et al., 2008). While binding of individual variable domains is modular, binding of whole Dscams is all-or-nothing (Wojtowicz et al., 2004; Wojtowicz et al., 2007; Sawaya et al., 2008). That is, the particular identities of the variable domains do not matter as long as they are the *same* between isoforms, as even a minor mismatch between one pair of variable domains is sufficient to totally disrupt binding (Wojtowicz et al., 2004; Wojtowicz et al., 2007; Sawaya et al., 2008).

Upon homophilic ectodomain binding, the C-terminal cytoplasmic tail of Dscam initiates repulsive signaling in the cell, which eventually leads to repulsion between cells expressing identical isoforms (Matthews et al., 2007). Although the mechanism that promotes this repulsion is still poorly understood, studies have identified a few conspicuous binding partners involved in cytoskeletal rearrangement (Hing et al., 1999; Schmucker et al., 2000; Worby et al., 2001). Among these is the adaptor protein Dock, which binds the Dscam cytoplasmic tail and recruits the effector kinase Pak1, which is implicated in several pathways underlying neurite repulsion (Figure 5A) (Hing et al., 1999). However, while the Dock-Pak axis seems to be necessary for Dscam-mediated axon guidance (Schmucker et al., 2000), loss of Dock or Pak1 does not produce dendrite self-crossing phenotypes (Hughes et al., 2007). This indicates that repulsive signaling required for Dscam-mediated self-avoidance can proceed independently of Dock and Pak.

A recent study investigating DSCAMs, the mammalian homologs of fly Dscams, found that both DSCAMs and Dscams share a predicted nuclear localization signal (NLS) and can be cleaved by proteolysis *in vivo* (Sachse et al., 2019). In mammalian DSCAMs, the NLS was found to promote translocation of cleaved DSCAM cytoplasmic fragments into the nucleus, where they affect expression of genes associated with synapse formation (Figure 5A) (Sachse et al., 2019). Future research should assess whether this signaling mode occurs in *Drosophila*.

### Mutually Exclusive Splicing Generates Isoform Diversity

Similar to their cousins in the immunoglobulin superfamily, Dscams rely on large arrays of diverse variants to function (Hattori et al., 2007). But unlike TCRs and BCRs, Dscam isoform diversity is generated at the RNA level. Schmucker and colleagues were the first to note the wide variety of Dscam isoforms in fruit flies (Schmucker et al., 2000). cDNA and genomic analyses of Bolwig’s nerves in *D. melanogaster* embryos revealed alternative sequences for the extracellular *Ig* domains 2, 3, and 7, as well as the transmembrane domain (Schmucker et al., 2000). The N-terminal half of Ig2 is encoded by variants of exon 4, the N-terminal half of Ig3 is encoded by variants of exon 6, the entire Ig7 domain is encoded by variants of exon 9, and the entire transmembrane domain is encoded by variants of exon 17 (Schmucker et al., 2000; Zhan et al., 2004). Exon clusters 4, 6, 9, and 17 were found to have 12, 48, 33, and 2 exon variants, respectively. Each variant is spliced in a mutually exclusive manner, such that each *Dscam* cDNA sequence only contains one of each variable exon 4, 6, 9, and 17 (Figure 4A) (Schmucker et al., 2000). Further, splicing of different exon clusters proceeds independently, which is why the *Dscam1* locus can combinatorially encode up to 38,016 unique isoforms (Schmucker et al., 2000). Combined with the fact that individual neurons express several isoforms simultaneously, *Dscam1* turns out to be a powerful system for uniquely “barcoding” cells (Celotto and Graveley, 2001; Neves et al., 2004; Zhan et al., 2004).

### Variant Exon Inclusion in Alternative Splicing Is Probabilistic

Based on data from *D. melanogaster* exon 4 splicing reporter lines, Miura and colleagues proposed that this mutually exclusive alternative splicing proceeds in a probabilistic fashion (Miura et al., 2013). Within class IV da neurons, they observed that all 12 variants of exon 4 had different yet stable probabilities of inclusion (Miura et al., 2013). In contrast, the probabilities of variant inclusion differed between different classes of neurons. For example, exon 4.2 was expressed in more than half of class IV da neurons, but negligibly expressed in Kenyon cells (Miura et al., 2013). Further, in comparing class IV da neurons in late second and wandering third instar larval stage, Miura and colleagues found that the inclusion frequencies of exon 4 variants changed over time (Miura et al., 2013). Their findings coincide with other studies which demonstrate that *Dscam1* exon selection is biased by developmental stage, tissue type, and even by neuronal subtype (Celotto and Graveley, 2001; Neves et al., 2004; Zhan et al., 2004).

Aside from the general observation that alternative splicing of exon clusters is probabilistic, the mechanism underlying exon variant selection remains to be elucidated; that is, it is still unclear how exon variants are specifically, exclusively, and stochastically selected for inclusion during splicing (Hemani and Soller, 2012). The picture is complicated by the fact that different exon clusters in the *Dscam1* locus seem to employ different methods for mutually exclusive alternative splicing (Hemani and Soller, 2012). To consider one proposed mechanism in detail, this review will focus on mutually exclusive splicing in the exon 6 cluster of the *D. melanogaster Dscam1* locus. More information on regulation of this or other clusters in *Drosophila* or other organisms can be found elsewhere (Graveley, 2005; Anastassiou et al., 2006; Yang et al., 2011; Wang S.-Z. et al., 2012; Hemani and Soller, 2012; Yue et al., 2016a; Yue et al., 2016b; Haussmann et al., 2019; Ustaoglu et al., 2019; Xu et al., 2019).

### RNA Secondary Structures Mediate Inclusion of Single Exon Variants

Similar to the selection mechanisms for *VSG*s and protocadherin exon variants (discussed below), the exon six variants of the *Dscam1* locus are maintained in a repressed state until a selection event specifically activates a variant for expression (Figure 4B). In the case of the *Dscam1* locus, the selection event is the formation of RNA secondary structures which antagonize binding of repressive heterogeneous nuclear ribonucleoproteins (hnRNPs) and promote binding of serine-arginine rich (SR) proteins (Olson et al., 2007).

Graveley (2005) first reported conserved sequences within the exon 6 cluster that seem to be required for mutually exclusive selection of exon six variants: a “docking site,” located in an intron upstream of the first exon six variant, and a “selector sequence,” one of which is located directly upstream of each of the 48 exon six variants (Graveley, 2005; Anastassiou et al., 2006; May et al., 2011). The docking site and each selector sequence are predicted to form an RNA stem-loop structure by base-pairing (Graveley, 2005; May et al., 2011). This stem-loop prevents splicing inclusion of the exon variants contained within the loop but promotes specific inclusion of the exon directly downstream of it (Graveley, 2005; Anastassiou et al., 2006; May et al., 2011; Hemani and Soller, 2012; Xu et al., 2019). Because the selector sequences bind to offset, overlapping portions of the docking site, only one selector sequence is predicted to bind, ensuring that there is only one such stem-loop structure at any given time (Graveley, 2005; Anastassiou et al., 2006; May et al., 2011). In addition to competing docking site-selector sequence interactions, it appears that a locus control region (LCR) is necessary for the activation of exon six variants (Wang X. et al., 2012). The LCR is a large tandem stem-loop RNA structure. In *Drosophila* species it forms a highly conserved “hexaleaf” consisting of ∼700 bp of scattered upstream intronic sequences (Wang S.-Z. et al., 2012).

RNAi screens by Graveley and colleagues identified Hrp36 (Hrb87F) as the hnRNP responsible for global repression of the exon 6 cluster (Olson et al., 2007). Hrp36 was shown to bind to the exon 6 cluster and is required to repress the inclusion of extra exon six variants. Further, Hrp36 was shown to inhibit binding of SR proteins, which are known to regulate alternative splicing and promote exon inclusion (Olson et al., 2007). Thus, the current model is that an Hrp36 protein binds at each exon six variant within the cluster, maintaining it in a repressed state until an upstream docking site-selector sequence stem-loop somehow dislodges Hrp36 from the proximal variant. The LCR may also help destabilize Hrp36 binding (Wang X. et al., 2012). Dissociation of the Hrp36 protein allows SR proteins to bind the proximal variant and promote its inclusion in splicing (Graveley, 2005; Olson et al., 2007; Hemani and Soller, 2012; Xu et al., 2019). Separately, it has also been suggested that the LCR promotes recognition of weak splice sites in exon variants. Specifically, upon formation of a docking site-selector sequence stem-loop, the LCR is brought within 100 bp of both splice sites of the proximal variant, allowing it to activate inclusion in a proximity-dependent manner (Wang S.-Z. et al., 2012).

To sum, exon inclusion in the *Dscam1* exon 6 cluster appears to be determined by the binding ability of different selector sequences, which may be modulated by splicing factors and RNA-binding proteins (RBPs) such as SR proteins and hnRNPs (or other, noncanonical RBPs, as in the case of the exon 9 cluster, reported elsewhere (Ustaoglu et al., 2019)). It is possible that *deterministic* regulation of these protein factors, which themselves mediate probabilistic events in splicing, underlies the “stochastic yet biased” expression of different exon variants among different cell types and at different times (Neves et al., 2004; Zhan et al., 2004; Miura et al., 2013). These protein factors may stably associate with splicing machinery in a complex with chromatin, allowing them to sterically exclude exon variants; this may also explain the fact that individual cells only express a finite number of Dscam isoforms (Miura et al., 2013). Future work should investigate the possibility of active negative feedback mechanisms regulating the number of expressed isoforms.

Experimental evidence and comparative genomic analyses indicate that selection of exons 4, 9, and 17 also relies on competing RNA secondary structures, which may be recognized by distinct but overlapping sets of RBPs. This is discussed further elsewhere (Park et al., 2004; Anastassiou et al., 2006; Olson et al., 2007; Yang et al., 2011; Wang X. et al., 2012; Hemani and Soller, 2012; Yue et al., 2016a; Yue et al., 2016b; Haussmann et al., 2019; Ustaoglu et al., 2019; Xu et al., 2019). There is still much to be understood about the mechanisms regulating exon choice within each cluster. It may also prove fruitful to investigate possible crosstalk between the different exon clusters.

### Dscam Diversity Is Required for Proper Neural Patterning

Studies that reduced the number of possible Dscams underline the importance of great isoform variety. Regarding self-avoidance, studies found that reducing the Dscam repertoire to just one isoform produced marked neural circuit defects in MB and olfactory receptor (OR) neurons (Hattori et al., 2007; Matthews et al., 2007; Soba et al., 2007). In another study that reduced the isoform repertoire, it was found that flies with at least 4,752 Dscam isoforms were indistinguishable from wild-type controls, while flies with 1,152 isoforms or less demonstrated substantial self-branching defects in da neurons (Hattori et al., 2009). These branching defects improved as the number of potential isoforms increased, indicating that self-avoidance requires several thousand different isoforms (Hattori et al., 2009). The finding that neurons require diverse Dscams not only to avoid synapsing with themselves but also to perform anatomic work such as axonal branching suggest that the repulsive force of self-avoidance is used to generate neuronal shapes. How the strength of this force is regulated or differentially harnessed in the production of distinct neuronal shapes is of interest in future work.

While a large variety of isoforms is clearly required, it is unclear whether any one isoform is necessary for normal patterning. In particular, studies that serially deleted different exon 4 variants (thereby eliminating particular Ig2 domains) did not produce observable phenotypes in MB or da neurons, indicating that self-avoidance does not require any specific isoform (Wang J. et al., 2004; Hattori et al., 2009). On the other hand, another study reducing diversity to 22,176 isoforms in mechanosensory neurons found defects in axonal branch extension and branching patterns that correlated with particular deletion alleles, suggesting that some connectivity patterns may be mediated by specific isoforms (Chen et al., 2006). It may be that specific isoforms are needed for some types of neural patterning processes, such as axonal targeting and branching, but not for dendritic self-avoidance. If so, this may also reconcile the bias for certain exon variants at certain developmental stages and within specific cell types: different Dscam isoforms may be required for different developmental and patterning processes (Celotto and Graveley, 2001; Zipursky and Sanes, 2010).

### Protocadherins as Analogs for Dscams

Vertebrate protocadherins function analogously to invertebrate Dscams in that both systems mediate neuronal self-avoidance (Garrett et al., 2018). As with invertebrate Dscams, diverse sets of protocadherin isoforms are generated and go on to mediate processes required for proper neural circuit wiring in vertebrates (Schreiner and Weiner, 2010). A notable difference between invertebrate Dscams and vertebrate protocadherins is how variation is produced in each system: alternative splicing generates diverse Dscam isoforms, while utilization of alternative promoters generates diverse protocadherin isoforms (Schmucker et al., 2000; Wojtowicz et al., 2004; Jin et al., 2018). Specifically, Dscams rely on splicing and associated proteins, while PCDHs utilize a CTCF/cohesin-mediated DNA looping mechanism to select proper promoters (Schmucker et al., 2000, p. 200; Wojtowicz et al., 2004). Both mechanisms, however, are prime examples of the ability of non-deterministic events to generate great protein diversity. Such diversity is especially helpful in patterning the nervous system, given that each neuron is likely to have several neighbors, each of which needs to have a different “barcode” if it is to form unique and overlapping networks of connections. Further, the binding of variable domains in either type of protein is exquisitely specific, much like the binding of immunoglobulins and T-cell receptors (Figure 5B) (Zipursky and Sanes, 2010). It is remarkable that Dscams and protocadherins have such convergent functions, given the significant differences in their phylogeny, morphology, and mechanistic origins.

While the *DSCAM* gene is conserved in mammals, it does not encode diverse isoforms. In some tissues, including the vertebrate eye, DSCAM has been shown to play a deterministic role in synaptic matching (Yamagata and Sanes, 2008). It has also been shown to increase stringency of synaptic partnerships in other areas of the brain (Garrett et al., 2018). However, a recent analysis found that mammalian DSCAM and DSCAML1, along with other members of the basigin-related family, are sometimes expressed in a mutually exclusive pattern (Iakovlev et al., 2021). It is therefore possible that *DSCAM* genes also contribute to non-deterministic aspects of cell identity in mammals.

## Protocadherins

The lack of Dscam diversity in mammals prompted a hunt for protein families performing barcoding or self-avoidance roles in mammals. Work over the last 20 years has shown that a subset of cadherins, the clustered protocadherins, mediates neuronal self-identification in mammals. Just like Dscams, protocadherins generate unique signatures, or “barcodes,” in individual neurons and function via homophilic repulsion (Weiner and Jontes, 2013). The regulation of clustered *PCDH* expression shares much in common with other systems discussed in this review: protocadherins utilize enhancers to stochastically select a promoter, similar to olfactory receptors; they contain variable and constant regions analogous to those of immunoglobulins; and their expression is sometimes monoallelic and restricted by heterochromatinization.

### Genomic Organization of Clustered Protocadherins

**FIGURE 6:**
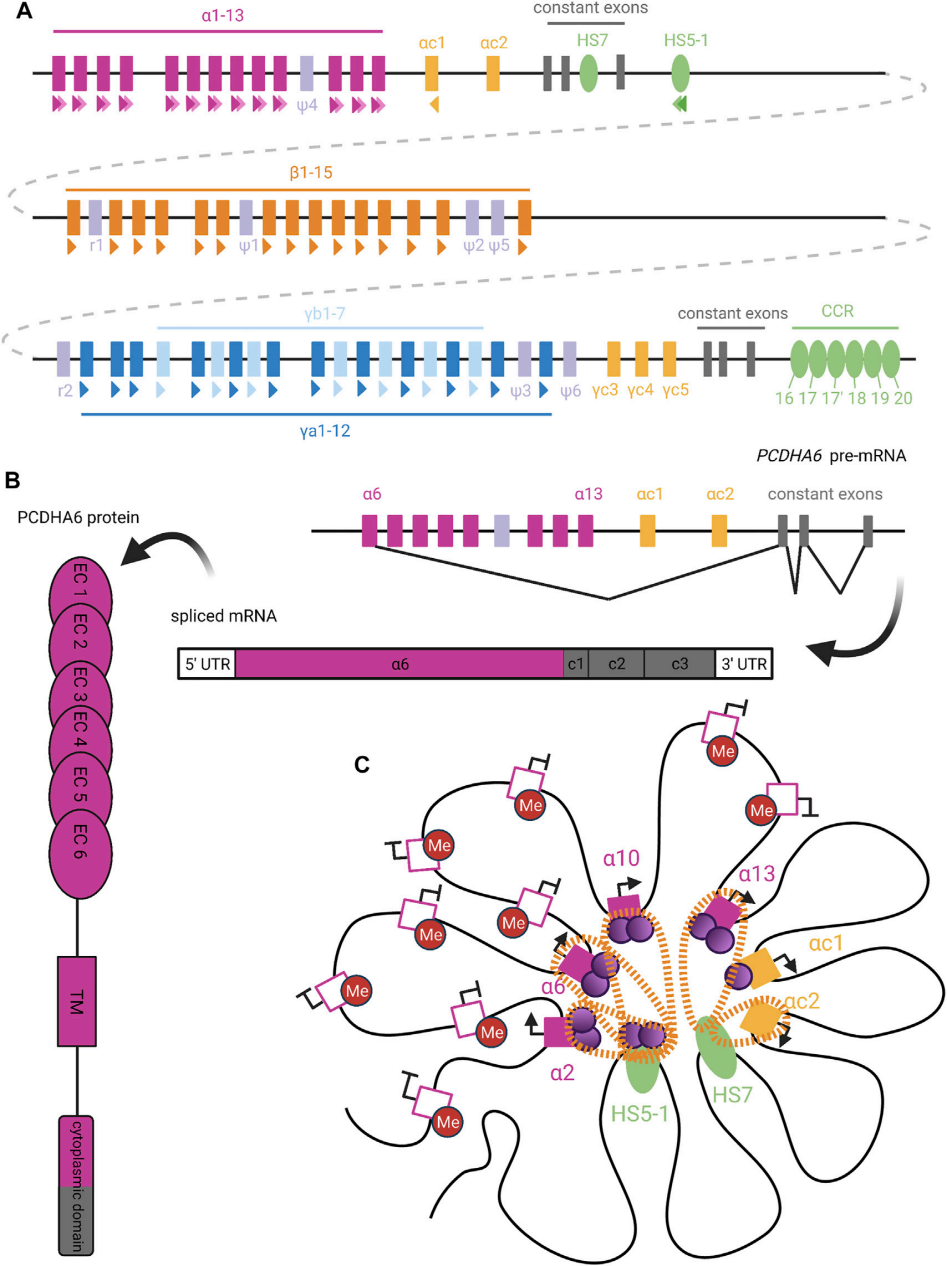
Organization and Expression of Protocadherins. **(A)** Genomic organization of the human clustered protocadherins. In humans, the *PCDHA*, *PCDHB*, and *PCDHG* genes are arranged in tandem clusters, with a few intervening pseudogenes (lavender), in the 5q31 region of chromosome 5 (Wu and Maniatis, 1999). The *PCDHA* and *PCDHG* clusters also have deterministically-expressed terminal V exons (yellow) and constant exons (grey), while the *PCDHB* cluster only contains stochastically-expressed V exons (Tasic et al., 2002). Colored triangles under exons or enhancers denote CSEs, with staggered triangles indicating two CSEs for a given element. **(B)** Upon *PCDHA* promoter selection, transcription proceeds through all downstream V and C exons. Intervening V exons are spliced out such that mature *PCDHA* mRNA contains just one variable exon spliced to three constant exons (Tasic et al., 2002). The variable exon encodes six ectodomains, the transmembrane domain, and the N-terminal half of the cytoplasmic domain in the PCDHA protein; the rest is encoded by the constant exons (Mah and Weiner, 2016). **(C)** Extensive DNA looping between active *PCDHA* promoters (filled pink or yellow squares) and long-range enhancers (HS5-1 and HS7; green ovals) is mediated by complexes of CTCF (purple circles) and cohesin (orange perforated bands) (Kehayova et al., 2011; Guo et al., 2012). In certain cases, binding of cohesin or CTCF alone (as with the *PCDHAC1* and *PCDHAC2* exons, respectively) is able to mediate promoter-enhancer interactions (Guo et al., 2012). To allow CTCF binding, candidate *PCDHA* genes must escape repressive DNA methylation (red circles), perhaps with the help of long non-coding RNA (Canzio et al., 2019). We diagram the potential for co-expression of a few isoforms in the same cell, as has been observed in single-cell analyses (Esumi et al., 2005; Kaneko et al., 2006; Mountoufaris et al., 2017). Figure inspiration was drawn from various sources (Sawaya et al., 2008; Weiner and Jontes, 2013; Massah et al., 2015; Mancini et al., 2020; Wu and Jia, 2021).

Clustered *PCDH*s are organized into alpha (*PCDHA*)*,* beta (*PCDHB*)*,* and gamma (*PCDHG*) clusters, arranged in tandem along chromosome 5 in humans. The *PCDHA* genes are encoded by a set of 15 large variable (V) exons that precede 3 constant (C) exons (Figure 6A) (Wu and Maniatis, 1999). Each V exon is preceded by its own promoter, and transcription can initiate from any of these 15 promoters. The first transcribed V exon, determined by which promoter is selected, is then spliced to the C exons, removing the intervening V exons. The *PCDHG* cluster is arranged similarly to the *PCDHA* cluster, with 22 V exons preceding 3 C exons (Morishita and Yagi, 2007). The *PCDHB* cluster differs from the other two in that it does not contain any C exons to complement its 22 V exons and is thus a set of distinct genes (Tasic et al., 2002).

The final 2 V exons within the *PCDHA* cluster, *PCDHAC1* and *PCDHAC2*, and the *PCDHGC3*, *PCDHGC4*, and *PCDHGC5* from the *PCDHG* cluster are very similar to each other but not to other V exons (Wu and Maniatis, 1999). These five exons are expressed deterministically and will not be discussed further here.

### Stochastic Selection of V Exon Promoters Dictates Isoform Expression

The promoters preceding *PCDH* V exons contain a conserved sequence element (CSE) (Wu et al., 2001), which has been found to serve as an essential promoter binding motif (Tasic et al., 2002). With the exception of *PCDHAC1* and *PCDHAC2*, a second CSE has been observed within the exonic sequence of each *PCDHA* V exon (Figure 6A) (Chen and Maniatis, 2013). Similar CSE regions are also present in transcriptional *cis*-enhancer elements that are specific to each *PCDH* gene cluster (Hirayama and Yagi, 2017): The *PCDHA* cluster enhancer element HS5-1 is found downstream of the cluster’s third C exon and contains two CSE sites (Ribich et al., 2006). A long-range *PCDHB* enhancer, known as the clustered control region (CCR), has also been identified downstream of the *PCDHG* cluster (Yokota et al., 2011). Deletion of these enhancers affected the expression of their respective clusters, with little effect on *PCDHG* cluster expression. This suggests the existence of distinct *PCDHG* enhancer element(s) (X*γ* elements) whose exact genomic location is unknown (Yokota et al., 2011).

In order to initiate transcription at a given V exon, the CSE sites within its promoter region, its respective cluster’s enhancer element(s), and exon sequence (for *PCDHA* only), must escape repressive methylation established by DNMT3B during embryogenesis (Garrett et al., 2019). A recent study has implicated antisense long non-coding RNA (as-lncRNA) in the demethylation of promoter and exonic CSE sites within the *PCDHA* cluster (Canzio et al., 2019). This process may facilitate variation in *PCDHA* promoter choice by equalizing interaction of the enhancer with distal and proximal promoters. That is, in the absence of methylation, proximal promoters are preferentially chosen (Canzio et al., 2019). Thus, global methylation of the alpha cluster, alleviated by stochastic demethylation by as-lncRNA, prevents inclusion bias caused by proximity of certain promoters to enhancer elements. The same study did not detect the presence of any as-lncRNA corresponding to the *PCDHB* and *PCDHG* clusters. As such, it remains unclear what mechanisms are responsible for the demethylation of CSE sites within these two clusters.

Once the appropriate CSE sites have been demethylated, the next step of transcription initiation can proceed. The CSEs serve as binding sites for the insulator protein CTCF, which can interact with cohesin to form a CTCF/cohesin complex (Guo et al., 2012). Chromosome conformation capture (3C) assays have demonstrated the ability of CTCF/cohesin complexes to mediate interactions between the V exon promoters and enhancers through DNA-looping (Figure 6C) (Guo et al., 2012). Interestingly, a second *PCDHA* enhancer element (HS7), which is located within the intron between the second and third C exons, lacks a CSE site but is still able to mediate DNA-looping through interaction with cohesin alone (Guo et al., 2012). Nonetheless, several studies have pointed towards CTCF/cohesin complex interactions between V exon promoters and enhancers as a necessary step for expression of most PCDH isoforms from all three clusters (Monahan et al., 2012; Hirayama and Yagi, 2017). This conclusion is further supported by the fact that in non-neuronal cell types, competitive binding of the REST/NRSF repressor complex to the HS5-1 enhancer, rather than the CTCF/cohesin complex, led to significantly decreased *PCDHA* expression (Kehayova et al., 2011). Unlike the proposed model for an “enhancer hub” similar to that involved in olfactory receptor choice, it remains unclear how transcription proceeds following the formation of these promoter/enhancer interactions (Guo et al., 2012).

Incredibly, *PCDH* genes are expressed both monoallelically and biallelically. All three *PCDH* clusters show monoallelic, combinatorial expression of the V exons (Esumi et al., 2005; Kaneko et al., 2006; Hirano et al., 2012). However, the 5 C-type variable exons of the alpha and gamma clusters, *PCDHAC1*, *PCDHAC2*, *PCDHGC3*, *PCDHGC4*, and *PCDHGC5*, are all expressed biallelically (Kaneko et al., 2006). Therefore, both the *PCDHA* and *PCDHG* clusters are regulated under different allelic gene regulation mechanisms, which may help to increase neuronal diversity (Kaneko et al., 2006).

Protocadherin expression is not fully monogenic—in at least some neuronal types, expression of >2 non-C-type isoforms together is common (Kehayova et al., 2011). This could result from unstable promoter choice, such that different isoforms are expressed sequentially and still present contemporaneously, or from incomplete dependence on exclusive enhancers. In support of the second model, PCDHA promoters display differential dependence on the HS5-1 enhancer; PCDHA1-5 promoters are only moderately affected by loss of HS5-1 and could be chosen by an additional enhancer (Kehayova et al., 2011). Remarkably, Dscam choice in insects is also non-exclusive. Mixing of isoforms, or indeed variation in chosen isoforms over time, should not impede the function of these gene families in self-recognition as long as all parts of the same neuron have the same isoform mix as one another.

### Roles in Neural Circuit Development

*PCDHG* genes are commonly studied and highly expressed in the dendrites of hippocampal neurons where homophilic cell-cell interactions between isoforms facilitate circuit complexity (Molumby et al., 2016). In these neurons, PCDHG acts locally to promote arborization via homophilic matching. This was shown by increasing the likelihood of PCDHG homophilic interactions using mutations, which subsequently increased dendritic and circuit complexity (Molumby et al., 2016). This is opposite of what would be expected in other cell types, like retinal starburst amacrine cells and Purkinje cells, in which homophilic PCDH interactions lead to a clear self avoidance pattern (Lefebvre et al., 2012). Thus, depending on the cell type, PCDHG interactions may lead to attraction, repulsion, or other dendritic arborization signaling events. It has also been shown that the *PCDHA* cluster is involved in these expression patterns and that PCDHA and PCDHG isoforms work synergistically to facilitate dendritic arborization pattern formation (Molumby et al., 2016; Ing-Esteves et al., 2018). Comparisons of an allelic series mutant support the conclusion that PCDHA and PCDHG function together in a dose-dependent and cell-type-specific manner to provide a critical threshold for PCDH activity. Although this does create a type of redundancy in *PCDH* stochastic expression, having both *PCDHA* and *PCDHG* expressed is critical for neuronal development (Ing-Esteves et al., 2018). PCDHB isoforms, similar to PCDHA and PCDHG, form *trans*-homophilic interactions (interactions with identical molecules on other cells), but expression patterns are not well classified (Yokota et al., 2011; Hasegawa et al., 2016).

### Structural Characterizations

Like Dscams, clustered protocadherins bind homophilically, and all the isoforms expressed in a cell must find a match in *trans* in order to induce strong enough binding to initiate downstream signaling cascades and cellular responses. Recent structural characterizations of the gene family show that the structure of the PCDH ectodomain is a zipper-like lattice formed by alternating *cis*- and *trans*-interactions (Figure 5B). As the protein extends out of the cell and interacts with the proteins of adjacent cells, a larger two-dimensional structure is created between the cell membranes (Brasch et al., 2019). This structure is the basis for the initial self-recognition step in neuronal self-avoidance (Brasch et al., 2019). Once these structures are formed, members of all three PCDH clusters can mediate highly specific homophilic recognition, maximizing the most favorable protein interactions. This favorable homophilic interaction is maximized when identical isoforms of clustered PCDHs are present. For example, neurons expressing five isoforms prefer to form homophilic aggregates with neurons expressing a full identical set of five isoforms rather than those expressing just three or four out of the five. Thus, self-recognition between different neurons is avoided by the expression of a single mismatched isoform (Thu et al., 2014). If there is a perfect match between isoforms (i.e. branches of the same Soma), repulsion of the neurite branches will occur to avoid overlapping. However, the downstream signaling mechanisms mediating repulsion are not yet understood.

## Olfactory Receptors

Similar to the protocadherin family, mammalian olfactory receptor genes are also expressed monoallelically and monogenically in neurons. Olfactory receptor (OR) genes are organized in large clusters in the genome and, like *VSG* genes or the *PCDHB* cluster, are each independent transcriptional units, not sets of overlapping possibilities like the *BCR*/*TCR*, *Dscam1*, and *PCDHA* and *PCDHG* loci. ORs are transmembrane chemoreceptors, found on the cell membranes of olfactory sensory neurons (OSNs), which recognize odorant molecules to detect smells. Buck and Axel correctly estimated that there are roughly 1,000 *OR* genes in the mammalian genome (Buck and Axel, 1991). Stochastic expression of individual *OR* genes across individual olfactory sensory neurons allows each neuron to serve as a sensor for a limited repertoire of odors, simplifying higher-order interpretation of olfactory information.

The mammalian olfactory system is composed of the main and accessory olfactory systems (Spehr and Munger, 2009). The main olfactory epithelium (MOE), located in the main olfactory system in the nasal cavity, contains the OSNs, which synapse onto the main olfactory bulb. The accessory olfactory system, which is located in the vomeronasal organ in the nasal cavity, contains OSNs that express vomeronasal receptors and is absent in humans and other primates. A further look into the *OR*s of mice and humans reveals that they share many subfamilies of *OR* genes. However, homology relationships are difficult to discern at the gene level, and mouse subfamilies typically include more *OR* genes than human subfamilies (Godfrey et al., 2004). This variation in the olfactory receptor repertoire occurs through a process of birth-and-death evolution, and species-level differences are thought to arise through a combination of adaptation to different niches and genetic drift (Nozawa and Nei, 2009). Selection on any one member of such a large gene family is expected to be weak.

Like Dscam isoforms, ORs are expressed through a biased stochastic process: While expression of each OR is restricted to a particular zone of the olfactory epithelium, within the zone each olfactory sensory neuron randomly expresses just one allele of one of the ∼1000 *OR* genes. Individual olfactory sensory neurons don’t “know” in advance which receptor they will express. This stochastic expression system may facilitate evolutionary turnover at the receptor level as newly born ORs can be expressed privately in existing cell types.

The axons of OSNs expressing the same receptor converge onto glomeruli in the olfactory bulb, which act as a functional unit of odor coding (Chesler et al., 2007). Since a given odorant activates multiple OR species and a given OR responds to multiple odorants, this results in received odorants producing unique combinations of activated glomeruli with varying magnitudes of activity in the olfactory bulb (Malnic et al., 1999). This ensures precise odorant perception and allows the mouse olfactory system, for example, to detect orders-of-magnitude more odors than the number of receptors encoded in the genome (Brochtrup and Hummel, 2011).

### Monoallelic and Monogenic Expression of ORs

**FIGURE 7:**
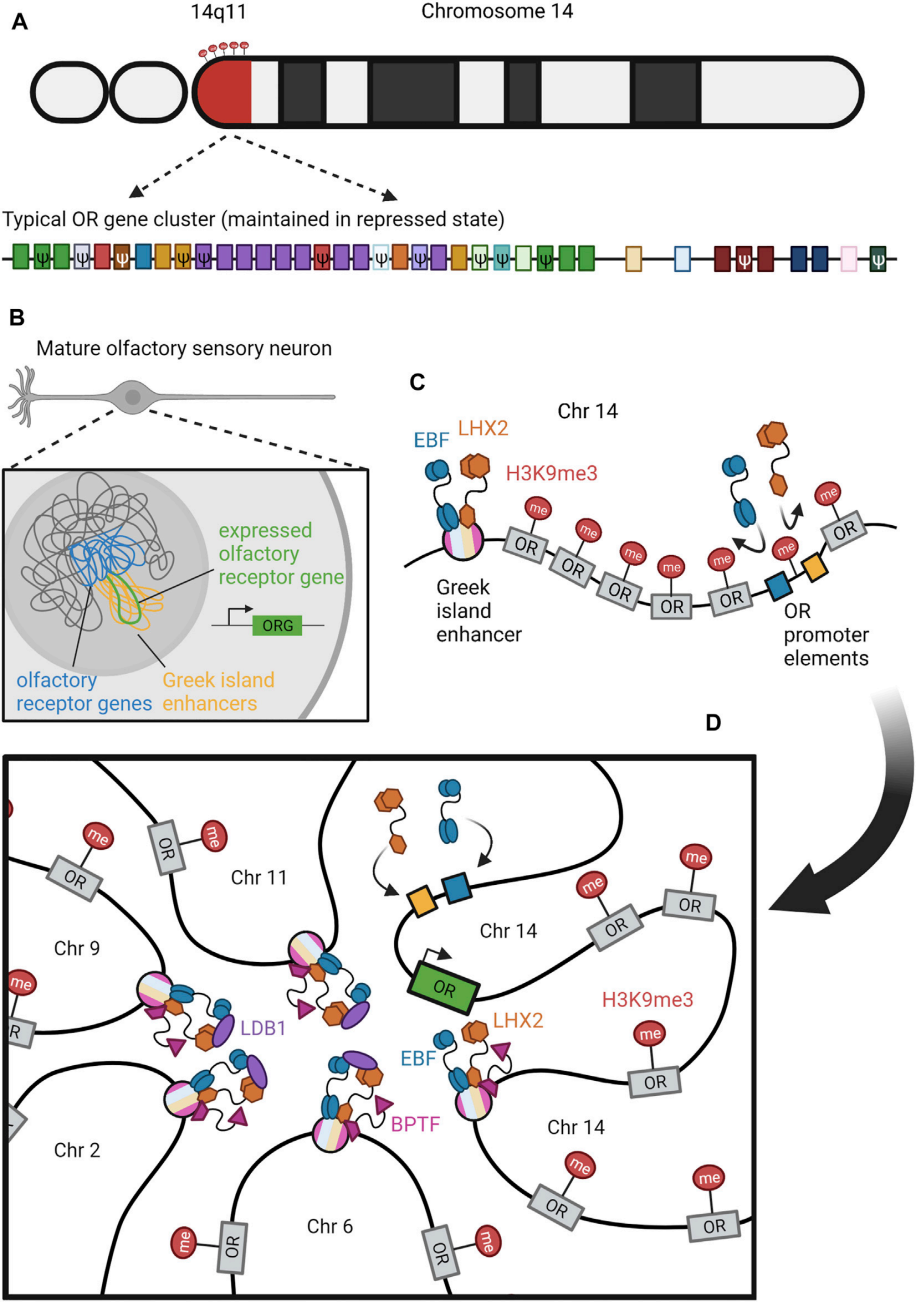
Organization and Expression of ORs. **(A)** Olfactory receptor (*OR*) genes are distributed throughout all chromosomes in the human genome except for chromosome 20. An example of a typical *OR* gene cluster, maintained in a repressed state by histone methylation, is shown on region 14q11 of chromosome 14. H3K9me3 is provided as an example of a heterochromatic modification (red lollipops). A close-up of this cluster reveals an array of functional and pseudo (ψ) *OR* genes belonging to various (color-coded) subfamilies (Olender et al., 2008). **(B)** In mature olfactory sensory neurons, *OR* genes are clustered into constitutive chromatin in the center of the nucleus (Clowney et al., 2012; Armelin-Correa et al., 2014; Armelin-Correa et al., 2015; Tan et al., 2019). Greek island enhancers (yellow strands) that are *cis* to inactive *OR* genes (blue strands) will form *trans* interactions to cluster around one active *OR* gene (green strand). Panel **(C,D)** detail the temporal progression leading to this arrangement. **(C)** Although Greek island enhancers can make *cis OR* genes competent for activation, they cannot completely relieve heterochromatic repression (red lollipops) (Serizawa et al., 2003; Nishizumi et al., 2007; Khan et al., 2011). Transcription factors BPTF (pink), EBF (blue), and LHX2 (orange) can bind to juxtaposed motifs within enhancers (striped circles), but are not strong enough to overcome heterochromatic repression of the same motifs in *OR* promoters (yellow and blue boxes) (Markenscoff-Papadimitriou et al., 2014; Monahan et al., 2017). **(D)** *Trans* interactions between Greek island enhancers on different chromosomes select one competent *OR* gene for activation (green box) (Markenscoff-Papadimitriou et al., 2014; Monahan et al., 2017; Monahan et al., 2019; Tan et al., 2019). Long-range, interchromosomal interactions are mediated by LDB1 (purple) in concert with EBF and LHX2 (Monahan et al., 2019). These interactions relieve repression on the promoter *cis* to the selected *OR*, allowing EBF and LHX2 to bind and promote transcription (Monahan et al., 2017). Figure inspiration was drawn from various sources (Malnic et al., 2004; Olender et al., 2008; Monahan et al., 2017; Bashkirova and Lomvardas, 2019; Monahan et al., 2019; Spitz, 2019) (Malnic et al., 2004; Olender et al., 2008; Monahan et al., 2017; Bashkirova and Lomvardas, 2019; Monahan et al., 2019; Spitz, 2019).

The expression of just a single olfactory receptor gene per OSN plays two critical roles in odor perception: First, it ensures that each OSN senses just a small set of odorant molecules; second, it allows olfactory sensory neurons to target specific glomeruli in the olfactory bulb. Multiple studies of differentiated OSNs have demonstrated that *OR* genomic regions remain intact throughout development (Eggan et al., 2004; Li et al., 2004). This is unlike the genetic recombination mechanisms we see in VSG and immunoglobulin diversity, which rearrange genomic loci. Further sequencing data confirmed that ORs are each encoded by individual genes rather than variable exons like *Dscam* and *Pcdh* genes (Li et al., 2004). Not only that, but a single *OR* gene is selected via the interaction of multiple enhancers (Figure 7), followed by feedback inhibition of remaining ORs (Monahan and Lomvardas, 2015). Investigations into *OR* selection have therefore focused on epigenetic and transcriptional regulators in an attempt to understand this stochastic system (Shykind, 2005).

In addition to being expressed monogenically (i.e. one OR per neuron), *OR*s are also expressed monoallelically. It is not clear if this is functionally important or if it is a side effect of the monogenic expression system. If the neuron treats all *OR* genes as identical and makes a single choice, then this would result in monoallelic as well as monogenic expression. However, previous studies have suggested that monoallelic silencing is a separate process from monogenic *OR* choice, and that it occurs earlier in development via DNA methylation (Chess et al., 1994; Armelin-Correa et al., 2014).

Transcription of multiple *OR* genes per OSN precedes the eventual translation of just a single OR per neuron (Hanchate et al., 2015; Tan et al., 2015). Immature neurons may express multiple receptors at low concentrations in overlapping regions of the nasal epithelium (Hanchate et al., 2015). This is reminiscent of the VSG system, in which cells may transcribe multiple *VSG*s before choice stabilization, and may implicate RNA-based mechanisms of gene silencing and/or feedback mechanisms informing the cell that successful translation has occurred.

### Heterochromatic Silencing of Non-chosen ORs

Despite coexpression of multiple *OR* genes in some immature OSNs, only one functional OR protein is translated in mature OSNs, and the rest of the gene family is transcriptionally silenced. Similar to many other surface molecules previously described, epigenetic regulation is essential for *OR* choice. Formation of heterochromatin throughout *OR* gene clusters likely occurs in an early stage preceding *OR* gene expression, although the extent of heterochromatic silencing in an OSN’s ground state is still undetermined (Magklara et al., 2011; Nagai et al., 2016). *OR* genes are marked by the constitutive heterochromatin marks H3K9me3 and H4K20me3, which is a common characteristic amongst other monoallelic expression patterns (Figures 7C,D) (Magklara et al., 2011). Histone methyltransferases G9a and GLP assist in H3K9 trimethylation and have been shown to be essential for promoting OR diversity, but their significance in the timeline of OSN development has not been explicitly explained (Ferreira et al., 2014; Lyons et al., 2014). Since *OR* genes are widely repressed by trimethylation, histone demethylases are required to remove constitutive repressive marks from heterochromatin in order to permit expression of the chosen *OR* (Magklara et al., 2011). The enzymes that demethylate each heterochromatin mark to its dimethylated state have yet to be discovered, but one H3K9me2 demethylase, LSD1, is required for *OR* transcription (Lyons et al., 2013).

Heterochromatinization of olfactory receptor loci likely allows their reorganization into dense nuclear aggregates and therefore prevents most alleles from contacting the transcriptional machinery (Magklara et al., 2011; Clowney et al., 2012; Le Gros et al., 2016). While heterochromatin is found at the periphery of most mammalian cell nuclei, OSNs and some other types of neurons display a peculiar architecture with constitutive heterochromatin concentrated at the center of the nucleus (Figure 7B) (Solovei et al., 2009; Clowney et al., 2012; Armelin-Correa et al., 2015; Tan et al., 2015). Moreover, *OR* genes from all of the chromosomal arrays coalesce into interchromosomal aggregates during OSN development (Clowney et al., 2012; Monahan et al., 2019; Tan et al., 2019). Dismantling these aggregates allows expression of multiple *OR*s per OSN, even when heterochromatin marks remain intact (Clowney et al., 2012). These aggregates are also known to be disrupted in COVID-induced anosmia (Zazhytska et al., 2021).

### Enhancer Activation

Reminiscent of *PCDH* promoter selection, enhancer activation is essential for *OR* gene expression. There is a large family of enhancers, called Greek islands, each serving a necessary function in activating their cluster of *cis OR*s (Serizawa et al., 2003; Nishizumi et al., 2007; Khan et al., 2011; Markenscoff-Papadimitriou et al., 2014). Additionally, these enhancers interact with one another in *trans* to form a large enhancer cluster that associates with a single *OR*, selecting it for activation (Figure 7D) (Markenscoff-Papadimitriou et al., 2014; Monahan et al., 2017; Monahan et al., 2019; Tan et al., 2019). These enhancer-*OR* clusters are specific to OSNs, and are not found in other olfactory epithelial cells (Tan et al., 2019). The enhancer regions contain homeodomain binding motifs that are essential for their regulatory function (Nishizumi et al., 2007; Monahan et al., 2019). In transgenic mice, the accumulation of these homeodomain binding motifs upstream of an *OR* promoter led to an increasing probability of reporter transcription (Vassalli et al., 2011). These observations suggest that the accumulation of multiple enhancers provides this homeodomain enrichment to promote *OR* transcription.

The dynamics of these clusters follow a pattern in which the *cis* interactions form first, and then aggregate with other *cis* clusters on separate chromosomes to form larger *trans* complexes (Figures 7C–D) (Monahan et al., 2019). The formation of the *OR* foci also plays an important role in bringing *trans* enhancers and *OR* genes together. As such, inhibition of *OR* foci leads to a decrease in enhancer cluster formation and association with *OR* genes, causing a decrease in *OR* expression (Clowney et al., 2012; Markenscoff-Papadimitriou et al., 2014). Recent single cell Hi-C analysis has revealed that the number of enhancers needed to activate a given *OR* is rather low compared to the total number available, with active *OR* genes being associated with an average of six enhancers (Tan et al., 2019).

Several integral proteins have been characterized as key facilitators of cluster formation and *OR* activation. BPTF and LDB1 bind the enhancers and facilitate the aggregation of large enhancer-*OR* complexes to promote *OR* expression (Markenscoff-Papadimitriou et al., 2014; Monahan et al., 2019). Additionally, LHX2 binds homeodomains (Hirota and Mombaerts, 2004; Rothman et al., 2005; Monahan et al., 2017; Monahan et al., 2019) and the Olf/EBF family bind O/E binding sites (Figures 7C–D) (Wang et al., 1997; Wang S. S. et al., 2004), both of which are found on the *OR* promoters and enhancers. The deletion of these transcription factors reduces *OR* expression, and the Olf/EBF family is tightly regulated by Ffp433/OAZ throughout OSN development (Cheng and Reed, 2007; Roby et al., 2012).

Taken together, these discoveries support a model in which each *OR* enhancer first associates in *cis* with a single *OR* gene to promote transcriptional competence, after which the nuclear localization of *OR* foci facilitates the aggregation of these *cis* enhancer-*OR* pairs into larger enhancer complexes (Bashkirova and Lomvardas, 2019). Stochastic association of this large enhancer complex with one of the *OR* genes selected by its *cis* enhancer then promotes its transcription via the accumulated recruitment of transcription factors. Several of these activating clusters emerge in a given OSN nucleus, necessitating a feedback mechanism to silence non-selected *OR* genes and stabilize the selected gene (Tan et al., 2019).

### Negative Feedback Regulation

In order to achieve proper OSN identity, *OR* gene silencing is necessary to ensure only one OR will function once the neuron has fully matured (Lewcock and Reed, 2004; Fleischmann et al., 2008; Zhou and Belluscio, 2012). Once a functional OR is translated and expressed at robust levels, a negative feedback mechanism called the unfolded protein response (UPR) is triggered (Dalton et al., 2013). During this response, the successful and abundant translation of an OR leads to downstream signaling that turns off important translation-initiation factors, ceasing translation of most other ORs while also inducing expression of adenylyl cyclase type 3 (ADCY3), a molecule that is essential for odor signaling (Ron and Walter, 2007; Wang S.-Z. et al., 2012; Dalton et al., 2013). This feedback mechanism is very similar to the process that takes place within B cells that confirms successful translation of a functional BCR (Hetz et al., 2020). Many chaperones are also essential for OR singularity by coordinating the UPR between organelles and the cell membrane (Ron and Walter, 2007; Sharma et al., 2017). Evidence from transgene studies suggests there are also *cis*-regulatory elements in the *OR* coding region that contribute to monogenic expression, but these elements have yet to be identified (Nguyen et al., 2007).

G protein signaling also plays a role in stabilizing *OR* choice following expression of the OR protein in the cell membrane. However, it is undetermined whether the chosen *OR* is the first to be expressed or the most robustly expressed. Nevertheless, the selected OR protein acts as a GPCR to activate G_olf_ to signal its presence as a functional OR. Specifically, the beta-gamma subunit of G_olf_ has been shown to contribute to *OR* gene silencing by heterochromatin regulation in zebrafish, while other model systems have pointed to the importance of the alpha subunit of G_olf_ and its mediation by RIC8B (Von Dannecker et al., 2006; Ferreira et al., 2014; Machado et al., 2017). Regardless of each subunit’s role, G_olf_ then activates ADCY3, which in turn downregulates the aforementioned histone demethylase LSD to prevent activation of other *OR* genes and stabilize *OR* choice (Imai et al., 2006; Dalton et al., 2013; Lyons et al., 2013). The opposing roles of ADCY3 and LSD1 are further reviewed in (Monahan and Lomvardas, 2015).

Altogether, there are multiple known mechanisms of *OR* gene expression and silencing that are essential for monoallelic and monogenic expression. Achievement of singularity has been shown to involve heterochromatin formation, nuclear compartmentalization, enhancer interactions, and a feedback inhibition pathway. However, the temporal and molecular overlaps between these pathways are not yet understood. More research is needed to outline the developmental timeline of *OR* expression and identify causal relationships between these mechanisms that ensure *OR* singularity. This diversity and singularity of *OR* expression facilitates the effective axon guidance and complex circuitry that allows us to detect such a wide spectrum of odors.

## Similarities and Differences Across Systems

The major shared feature of the five systems covered in this review is their selection of a specific genetic option among a collection of many alternatives in order to express a surface protein that is integral to the cell’s identity. While each system has evolved unique mechanisms of monogenic and/or monoallelic expression, there are many parallels in the strategies adopted to accomplish selection, expression, and stabilization of the selected sequence (Table 1).

First, generation of combinatorial products is a key feature in many of these systems that expands the number of potential surface molecules relative to the number of coding segments encoded in the genome. The antigen receptors utilize V(D)J recombination, clustered *PCDH* genes select variable segments, and *Dscam1* splicing combines variable exons to create a massive repertoire of possible combinations from a small number of gene segments. *VSG* genes similarly combine pre-existing *VSG* gene segments to create new mosaic *VSG*s, and, unlike the other systems, these repertoires are constantly evolving and growing as new *VSG*s are made and stored.

These systems also exhibit context-driven restrictions and biases regarding which genes or gene segments are chosen. Within the olfactory epithelium, OSNs are spatially segregated into different zones, and each OR is expressed only within a single zone. Dscams and PCDHs show a similar restriction, but based on cell type rather than spatial location, as the pattern of variable domains expressed appears to vary between different populations of neurons. *VSG* expression instead follows a temporal bias for which the probability of certain VSGs appears to change throughout the course of infection, with mosaic VSGs emerging in the later stages of infection. Within the confines of these spatial, cell type, and temporal restrictions the available genes and gene segments still seem to be stochastically distributed. The mechanisms that restrict stochasticity are largely unknown.

The process determining which *VSG* expression site is active relies heavily on nuclear localization and chromatin remodeling, a feature shared by the other four systems. During VSG, OR, and antigen receptor selection the inactive alleles are repressed in heterochromatic regions, with *VSG* and antigen receptor loci localized to the nuclear periphery and *OR* gene arrays compacted into repressive foci. The selected allele in these systems is found in accessible euchromatic regions, which for antigen receptors is in the nuclear center, for *OR*s entails the active gene escaping the repressive foci, and for *VSG* genes requires the single active expression site to localize to the expression body. These nuclear localization events are intimately tied to chromatin modifications and DNA methylation. *VSG* expression sites localized to the expression body show significant nucleosome depletion. Interestingly, the active *VSG* expression site is enriched with H3K10 acetylation as well as H3K4 trimethylation. H3K4me3 is an activating signature that is also found in the active regions of the OR, *PCDH*, and antigen receptor loci. Histone modifications are prominent features of these systems. The antigen receptor loci are further activated by H3K9 acetylation and H3K36 trimethylation, whereas the inactive locus is enriched for repressive H3K9 di- and trimethylation. H3K9 methylation is also utilized to repress *OR* loci alongside H4K20 di- and trimethylation.

Singular *cis*-acting elements are also a common feature activating the selected sequences throughout many of these systems. Antigen receptors and *PCDH* loci share mechanistically similar downstream enhancer elements that loop back to the loci and activate the selected promoter, in both cases scanning for the first available promoter sequence either at the most proximal V segment or hypomethylated *PCDH* segment. Enhancers play a role not only in inducing expression of the selected *OR* gene, but also in carrying out the selection process, as enhancers coalesce first in *cis*, and then in *trans* onto a given *OR* gene for selective expession. Even though Dscam selection occurs at the RNA level, an enhancer-like element (the docking site-selector sequence stem-loop) occurs in the secondary structure of the transcript. Reminiscent of the previous systems, the stochastic binding of variant selector sequences to the docking site (which forms the stem-loop) dictates the pattern of exon splicing. While *VSG* expression has not been shown to require enhancer interactions, the selected *VSG* still must be located within the active expression site. Further research into whether a limiting enhancer selects a *VSG* for selection at a particular time will be of great interest.

## Conclusion

In this review, we have emphasized the importance of stochastic processes in achieving variability in barrier and nervous systems. By analyzing immune system evasion by the parasite *Trypanosoma brucei*, immune system pathogen identification by B cell and T cell receptors, neuronal self-avoidance through diverse expression and self-binding processes of Dscams and protocadherins, and the perception of stimuli through the olfactory system, we have shown that the role of diversity is crucial to the survival and function of organisms through both internal and environmental interactions. Common processes such as monoallelic expression, epigenetic regulation, and specificity of binding of variable domains were discussed. It is important to recognize that all five systems studied are examples of surface molecules that utilize the stochastic selection of genetic material to create cellular diversity. Like aleatoric music and the world-generating algorithms of sandbox games, these systems maximize limited genetic information to construct complex and unpredictable outcomes.
